# New species and new records of *Trechispora* (Trechisporales, Basidiomycota) from Taiwan

**DOI:** 10.1186/s40529-025-00469-9

**Published:** 2025-07-21

**Authors:** Yi-Chung Lin, Yu-Ming Huang, Yi-Lun Huang, Shi-Liang Liu, Shuang-Hui He, Li-Wei Zhou, Che-Chih Chen

**Affiliations:** 1 Department of Biology, National Museum of Natural Science, Taichung, 404605 Taiwan; 2 Department of Plant Pathology, National Chung Hsing University, Taichung, 402202 Taiwan; 3 State Key Laboratory of Microbial Diversity and Innovative Utilization, Institute of Microbiology, Chinese Academy of Sciences, Beijing, 100101 China; 4 Institute of Microbiology, School of Ecology and Nature Conservation, Beijing Forestry University, Beijing, 100083 China

**Keywords:** 4 new taxa, Coral fungi, Corticioid fungi, Hydnodontaceae, Systematics

## Abstract

**Background:**

*Trechispora* (Hydnodontaceae) comprises a diverse group of wood- and soil-inhabiting fungi, primarily functioning as saprotrophs, with some species forming symbiotic associations with plants and animals. Despite the recognition of over 100 species worldwide, its diversity in Taiwan remains understudied. This study presents the first comprehensive taxonomic revision of *Trechispora* in Taiwan, integrating morphological and phylogenetic analyses based on sequence data from the nuc rDNA internal transcribed spacer ITS1-5.8S-ITS2 (ITS) region and the nuc 28S rDNA (28S).

**Results:**

We describe four new species (*Trechispora acerosa*, *T. floralis*, *T. formosana*, and *T. orchidophila*) and report seven newly recorded species for Taiwan (*T. crystallina*, *T. dentata*, *T. latehypha*, *T. mollusca*, *T. odontioidea*, *T. subsinensis*, and *T. wenshanensis*). *T. sinensis* is synonymized under *T. odontioidea*. Morphological and phylogenetic analyses support their taxonomic placements, and an identification key to accepted *Trechispora* species in Taiwan is provided.

**Conclusion:**

This study expands the known diversity of *Trechispora* in Taiwan to 17 species, highlighting their ecological significance and potential interactions with plants in Taiwan’s forest ecosystems.

## Background

*Trechispora* (Hydnodontaceae Jülich) is the largest genus in Trechisporales K.H. Larss (Hibbett et al. 2007), typified by *T. onusta* P. Karst. (Karsten 1890). It includes basidiomata of coralloid, pileate, and resupinate forms, with smooth, grandinioid, odontioid, hydnoid, or poroid hymenophores (Bernicchia and Gorjón 2010; de Meiras-Ottoni et al. 2021; Karsten 1890). Microscopically, *Trechispora* has monomitic or dimitic hyphal systems with nodose-septate generative hyphae, short basidia, and basidiospores ranging from smooth to verrucose or aculeate. Ampullate septa occur on subicular hyphae, especially in some mycelial cords (Larsson 1992). Calcium oxalate crystals on the subicular hyphae provide additional diagnostic features (Larsson 1992, 1994).

Most *Trechispora* species are saprotrophs found on decayed wood, bamboos, grasses, palms, tree ferns, mosses, or soil with litter (Chikowski et al. 2020; Larsson 1992; Lin et al. 2022; Liu et al. 2019). They play an important role as decomposers in forest ecosystems (de Meiras-Ottoni et al. 2021; Jülich 1976; Larsson 1992). Some species have been identified as potential ectomycorrhizal fungi (Dunham et al. 2007; Rosenthal et al. 2017; Vanegas-Leon et al. 2019), endophytes (Fulthorpe et al. 2020; Shen et al. 2022), or even pathogens (Wilkinson 1987). Additionally, Matsuura and Yashiro (2010) reported an unknown *Trechispora* species forming termite-mimicking sclerotia in *Nasutitermes takasagoensis* nests.

Currently, over 100 species of *Trechispora* are recognized worldwide (Chikowski et al. 2020; Drechsler-Santos et al. 2008; Hjortstam and Ryvarden 2007; Kirk et al. 2008; Larsson 1994, 1996; Liu et al. 2022a, 2022b; Luo and Zhao 2022; Ordynets et al. 2015; Phookamsak et al. 2019; Westphalen and Silveira 2012). Among these, only seven species have been recorded in Taiwan: *T. cryptomerioides* (W.-R. Lin & P.-H. Wang) S.L. Liu & L.W. Zhou, *T. dimitica* Hallenb., *T. farinacea* (Pers.) Liberta, *T. lunata* (Romell ex Bourdot & Galzin) Jülich, *T. praefocata* (Bourdot and Galzin) Liberta, *T. rigida* (Berk.) K.H. Larss., and *T. taiwanensis* S.L. Liu, S.H. He & L.W. Zhou (Lin et al. 2022; Liu et al. 2022a; Maekawa 1992; Wu 2003).

This study provides the first comprehensive taxonomic revision of *Trechispora* in Taiwan, integrating morphological and phylogenetic analyses. We describe and illustrate newly discovered species, clarify the taxonomic placement of previously recorded taxa, and present an identification key to accepted *Trechispora* species in Taiwan.

## Materials and methods

### Morphological studies

The studied specimens were deposited at the fungaria of Beijing Forestry University, Beijing (BJFC), and the National Museum of Natural Science, Taichung (TNM). The acronyms follow the Index Herbariorum (http://sweetgum.nybg.org). Basidiomata were photographed using an Olympus TG-4 or a Canon EOS R digital camera. The hymenial surface was examined under an Olympus SZ51 (Olympus, Tokyo, Japan) stereomicroscope at magnifications up to × 50. Free-hand thin sections of basidiomata were mounted in 5% potassium hydroxide (KOH) with 1% phloxine for observation and measurements and in Melzer’s reagent to test for amyloidity and dextrinoidity, using a ZEISS Axioscope 5 microscope (Carl Zeiss, Germany) at magnifications up to 1000 × . The following abbreviations are used: L = mean basidiospore length (arithmetical average for all basidiospores), W = mean basidiospore width (arithmetical average for all basidiospores), Q = L/W ratio, n = the number of basidiospores measured per specimen, m = meters above sea level. Microscopic measurements and morphological terminology followed the protocols established by Wu (1990).

For scanning electron microscopy (SEM) imaging, small fragments of dried basidiomata were directly mounted onto specimen stubs. The samples were then sputter-coated with platinum using a JEC-3000FC Auto Fine Coater (JEOL, Tokyo, Japan) and examined for basidiospore and crystal morphology using a JSM-7800F Schottky Field Emission Scanning Electron Microscope (JEOL).

### DNA extraction, polymerase chain reaction (PCR), and DNA sequencing

DNA was extracted from either dried specimens or mycelia cultivated on 2% MEA. Tissue disruption and homogenization were performed before extraction with the aid of liquid nitrogen and Tissue Lyser II (Qiagen, Hilden, Germany). DNA was extracted using the NautiaZ Plant DNA Extraction Mini Kit (Nautia Gene, Taipei, Taiwan) following the manufacturer’s protocols. The study targeted two genetic regions: the nuc rDNA internal transcribed spacer ITS1-5.8S-ITS2 (ITS) region was amplified using the primer pair ITS1F/ITS4B (Gardes and Bruns 1993), while the nuc 28S rDNA (28S) was amplified using primer pair LR0R/LR5 (Vilgalys and Hester 1990). Amplifications were conducted in a Bio-Rad T100™ Thermal Cycler (Bio-Rad, Hercules, California, U.S.). The PCR conditions for the ITS and 28S regions were as follows: initial denaturation at 94 °C for 5 min, followed by 36 cycles at 94 °C for 30 s, 55 °C for 30 s, and 72 °C for 1 min, and a final extension at 72 °C for 7 min. PCR products were purified and sequenced using the Sanger method by Genomics BioSci & Tech (New Taipei, Taiwan). Newly generated sequences were manually assembled using BioEdit v. 7.2.5 (Hall 1999) or Geneious Prime 2024.0.5 (https://www.geneious.com), and their quality was checked based on five guidelines by Nilsson et al. (2012) before submission to GenBank (https://www.ncbi.nlm.nih.gov/genbank/). Consensus sequence accuracy and identity were verified by comparison with sequences in GenBank.

### Alignment, phylogenetic analyses, and visualizing trees

Sequences of each single-gene dataset were aligned with MAFFT v. 7.409 (Katoh and Standley 2013), using the default algorithm and manually adjusted with MEGA v. 7 (Kumar et al. 2016) when necessary. *Porpomyces mucidus* (Pers.) Jülich was selected as the outgroup according to Liu et al. (2022a). The resulting alignments were deposited at Figshare (http://dx.doi.org/10.6084/m9.figshare.28407632). The Bayesian Inference (BI) and Maximum Likelihood (ML) methods were applied to the datasets using MrBayes v. 3.2.6 (Ronquist et al. 2012) and RAxML Black-Box (Stamatakis 2014), respectively. For the BI analyses, jModeltest 2.1.10 (Darriba et al. 2012) was first used to estimate separate models for each gene region in all datasets, based on Akaike information criterion (AIC). The Markov chain Monte Carlo (MCMC) search was run for ten million generations, with four chains and trees sampled every 1000 generations. The first 25% of trees (2500) were discarded as burn-in while the remaining trees were used to construct the 50% majority-rule consensus phylogram with posterior probabilities (PP). For the ML analysis, the best-scoring tree with proportional values of bootstrap (BS) was computed under a GTRGAMMA model with 1000 bootstrap replicates, followed by a thorough ML search. Partitioned BI and ML analyses were conducted (Table 1). Gaps were treated as missing data. Branches were regarded as having statistical support if values of PP and/or BS were ≥ 0.9 and ≥ 70%, respectively. Both BI and ML analyses were performed at the CIPRES Science Gateway (Miller et al. 2010; https://www.phylo.org/). The phylogenetic trees were visualized and edited in TreeGraph2 (Stöver and Müller 2010), Interactive Tree of Life (iTOL) v6 (Letunic and Bork 2024; https://itol.embl.de/), and Adobe Illustrator 27.9 (Adobe Systems, Inc).

**Table 1:** Species and sequences used in the phylogenetic analyses

Species	Sample no	Location	ITS	28S	References
*Dextrinocystis calamicola*	He 5693*	China	MK204533	MK204546	Liu et al. (2019)
*Porpomyces mucidus*	Dai 12692*	Czech Republic	KT157833	KT157838	Wu et al. (2015)
***Trechispora acerosa***	Chen 3186*	**Taiwan**	**PV085797**	**PV085828**	**This study**
***T. acerosa***	**GC 1612-34**	**Taiwan**	**PV085798**	**PV085827**	**This study**
*T. acerosa*	He 4641	China	OM523513	OM339319	Liu et al. (2022a)
*T. alba*	CH21384*	China	OR557258	–	Liu et al. (2024)
*T. alba*	HG 19350	China	OM523516	–	Liu et al. (2022a)
*T. albofarinosa*	CLZhao 4356	China	OQ241383	OQ282703	Luo et al. (2024)
*T. alnicola*	AFTOL-ID 665	–	DQ411529	AY635768	Unpublished
*T. araneosa*	KHL 8570	Sweden	AF347084	AF347084	Larsson et al. (2004)
*T. bambusicola*	CLZhao 3302	China	MW544021	MW520171	Zhao and Zhao (2021)
*T. bambusicola*	CLZhao 3305*	China	MW544022	MW520172	Zhao and Zhao (2021)
***T. bambusicola***	**Wu 9508-258**	**China**	**PV085818**	**PV085820**	**This study**
*T. bispora*	CBS:142.63	Australia	MH858241	MH869842	Vu et al. (2019)
*T. bisterigmata*	CLZhao 2522*	China	OQ241386	–	Luo et al. (2024)
*T. candidissima*	Dai 7092	China	OM523407	OM339229	Liu et al. (2022a)
*T. caucasica*	O-F-253764	Sweden	UDB038261	–	Unpublished
*T. caulocystidiata*	FLOR 56314*	Brazil	MK458772	–	Furtado et al. (2021)
*T. chaibuxiensis*	LWZ 20170814-34*	China	OM523409	OM339231	Liu et al. (2022a)
*T. chartacea*	FLOR 56185	Brazil	MK458775	–	Liu et al. (2022a)
*T. clancularis*	FRDBI 4426619	UK	MW487976	–	Unpublished
*T. cohaerens*	HHB 19445	New Zealand	MW740327	–	Unpublished
*T. confinis*	KHL 11064	Sweden	AF347081	AF347081	Larsson et al. (2004)
*T. constricta*	Dai 10534*	China	OM523416	–	Liu et al. (2022a)
*T. constricta*	He 5899	China	OM523417	OM339236	Liu et al. (2022a)
*T. copiosa*	AMO422*	Brazil	MN701013	MN687971	de Meiras-Ottoni et al. (2021)
*T. cryptomerioides*	0906RK10-23	Taiwan	KF679506	OK422242	Lin et al. (2022)
***T. cryptomerioides***	**GC 2308-51**	**Taiwan**	**PV085817**	**–**	**This study**
*T. crystallina*	LWZ 20171013-7*	Vietnam	OM523420	OM339239	Liu et al. (2022a)
***T. crystallina***	**WEI 20-056**	**Taiwan**	**PV085799**	**–**	**This study**
*T. cyatheae*	FR 0219443*	Franch	UDB024016	UDB024017	Ordynets et al. (2015)
*T. damansaraensis*	LWZ 20180417-26*	Malaysia	–	OM339241	Liu et al. (2022a)
*T. damansaraensis*	He 6415	Malaysia	OM523421	OM339240	Liu et al. (2022a)
*T. dealbata*	FLOR 56183	Brazil	MK458777	–	Liu et al. (2022a)
***T. dentata***	**Chen 4606**	**Taiwan**	**–**	**PV085826**	**This study**
*T. dentata*	Dai 22565*	China	OK298491	OM049408	Liu et al. (2022b)
*T. dimitica*	FRDBI 13394362	UK	MW487977	OR892779	Unpublished
*T. dimitiella*	Dai 21181	China	OK298493	OK298949	Liu et al. (2022b)
*T. echinocristallina*	FR 0219445*	Franch	UDB024018	UDB024019	Ordynets et al. (2015)
*T. echinospora*	MA-Fungi 82485	Equatorial Guinea	JX392845	JX392846	Tellería et al. (2013)
*T. farinacea*	KHL 8451	–	AF347082	AF347082	Unpublished
*T. fimbriata*	CLZhao 4154	China	MW544023	MW520173	Zhao and Zhao (2021)
*T. fissurata*	LWZ 20171015-35*	Vietnam	OM523431	OM339249	Liu et al. (2022a)
***T. floralis***	Wu 1703-66*	**Taiwan**	**PV085813**	**PV085822**	**This study**
*T. foetida*	FLOR 56315*	Brazil	MK458769	–	Furtado et al. (2021)
***T. formosana***	Chen 3151*	**Taiwan**	**PV085800**	**PV085823**	**This study**
*T. fragilis*	Dai 20535*	China	OK298494	OK298950	Liu et al. (2022b)
*T. gelatinosa*	AMO1139*	Brazil	MN701021	MN687978	de Meiras-Ottoni et al. (2021)
*T. gracilis*	LWZ 20210626-5b*	China	OM523436	OM339254	Liu et al. (2022a)
*T. havencampii*	SFSU DED8300*	Africa	NR154418	KT253947	Desjardin and Perry (2015)
*T. hondurensis*	HONDURAS19-F016*	Honduras	MT571523	MT636540	Unpublished
*T. hymenocystis*	Dai 2247	Finland	OM523439	–	Liu et al. (2022a)
*T. hymenocystis*	KHL 8795	Sweden	AF347090	AF347090	Larsson et al. (2004)
*T. incisa*	GB-0090648	Sweden	KU747095	KU747087	Unpublished
*T. invisitata*	UC2023088	USA	KP814425	–	Unpublished
*T. kavinioides*	KGN 981002	Norway	AF347086	AF347086	Larsson et al. (2004)
*T. khokpasiensis*	MEL2382623	Australia	KP012986	–	Deng et al. (2023)
*T. khokpasiensis*	MMCR00009*	Thailand	MZ687107	MZ683197	Deng et al. (2023)
*T. khokpasiensis*	ZP-1029	China	OM523532	–	Deng et al. (2023)
*T. laevis*	TUF115551	Estonia	UDB016406	–	Unpublished
*T. laevispora*	Dai 21655*	China	OK298495	OM108710	Liu et al. (2022b)
*T. larssonii*	LWZ 20190817-11a*	China	OM523442	OM339259	Liu et al. (2022a)
***T. latehypha***	**Chen 3372**	**Taiwan**	**PV085801**	**–**	**This study**
***T. latehypha***	**GC 2109-57**	**Taiwan**	**PV085802**	**–**	**This study**
*T. latehypha*	He 3924	China	OM523443	OM339261	Liu et al. (2022a)
*T. latehypha*	He 5438*	China	OM523445	–	Liu et al. (2022a)
*T. laxa*	MHHNU10714*	China	OP959650	OP954661	Deng et al. (2023)
*T. longiramosa*	CH 19233*	China	OM523449	–	Liu et al. (2022a)
*T. longiramosa*	HG 140168	China	OM523448	OM339264	Liu et al. (2022a)
*T. malayana*	Dai 17876*	Singapore	OM523452	OM339265	Liu et al. (2022a)
*T. mellina*	URM85756	Brazil	–	MH280000	Chikowski et al. (2020)
*T. microspora*	FRDBI 18772216	UK	OL828778	–	Unpublished
*T. minispora*	AM176	Mexico	MK328886	MK328895	Yuan et al. (2020)
*T. mollis*	URM85884*	Brazil	MK514945	MH280003	Chikowski et al. (2020)
***T. mollusca***	**Chen 2422**	**Taiwan**	**PV085803**	**–**	**This study**
*T. mollusca*	Dai 6191	China	OM523455	OM339269	Liu et al. (2022a)
*T. murina*	CLZhao 11752*	China	OL615004	OL615009	Luo and Zhao (2022)
*T. nivea*	LWZ 20180804-3	China	OM523461	OM339273	Liu et al. (2022a)
*T. orchidophila*	FM151.1	Réunion, Franch	JF691276	–	Martos et al. (2012)
***T. orchidophila***	Wu 1703-55*	**Taiwan**	**PV085812**	**PV085824**	**This study**
*T. orchidophila*	Y453-2	Okinawa, Japan	LC327027	–	Ogura‐Tsujita et al. (2018)
***T. odontioidea***	**Chen 1371**	**Taiwan**	**PV085804**	**–**	**This study**
***T. odontioidea***	**Chen 3891**	**Taiwan**	**PV085805**	**–**	**This study**
*T. odontioidea*	CLZhao 17890*	China	ON417458	OQ282713	Luo and Zhao (2022)
***T. odontioidea***	**GC 1602-4**	**Taiwan**	**PV085806**	**PV085829**	**This study**
***T. odontioidea***	**GC 1612-33**	**Taiwan**	**PV085807**	**–**	**This study**
***T. odontioidea***	**GC 1612-37**	**Taiwan**	**PV085808**	**PV085830**	**This study**
***T. odontioidea***	**GC 1703-113**	**Taiwan**	**PV085809**	**PV085819**	**This study**
***T. odontioidea***	**GC 1704-36**	**Taiwan**	**PV085810**	**–**	**This study**
*T. olivacea*	CLZhao 17826	China	ON417457	OQ282714	Luo and Zhao (2022)
*T. pallescens*	SC1	India	MZ518207	MZ518091	Unpublished
*T. papillosa*	AMO795	Brazil	MN701023	MN687981	de Meiras-Ottoni et al. (2021)
*T. patawaensis*	VPapp-GF1901*	French	OL314550	OL314546	Crous et al. (2021)
*T. perminispora*	LWZ20190816-39a*	China	OM523525	OM339329	Liu et al. (2022a)
*T. praefocata*	FRDBI 18819116	UK	OL828784	–	Unpublished
*T. regularis*	KHL 10881	Jamaica	AF347087	AF347087	Larsson et al. (2004)
*T. rigida*	URM85754	Brazil	MT406381	MH279999	Chikowski et al. (2020)
*T. robusta*	FLOR 56179	Brazil	MK458770	–	Furtado et al. (2021)
*T. saluangensis*	MMCR00260*	Thailand	MZ687104	MZ683201	Sommai S et al. 2023
*T. sanpapaoensis*	MEL:2382675	Australia	KP013038	–	Unpublished
*T. sanpapaoensis*	MMCR00124.1	Thailand	MZ687109	MZ683200	Sommai et al. (2023)
*T. scabra*	FLOR 56189	Brazil	MK458773	–	Furtado et al. (2021)
*T. sinensis*	LWZ 20180804-19*	China	OM523482	OM339290	Liu et al. (2022a)
*Trechispora* sp.	AMO799	Brazil	MN701008	MN687969	de Meiras-Ottoni et al. (2021)
*Trechispora* sp.	BAB5120	India	KT804576	–	Unpublished
*Trechispora* sp.	Dai 16179	China	OM523506	OM339313	Liu et al. (2022a)
*Trechispora* sp.	Dai 18781	Australia	OM523508	OM339315	Liu et al. (2022a)
*Trechispora* sp.	Dai 22173	China	OK298496	OK298951	Liu et al. (2022b)
*Trechispora* sp.	Dai 22174	China	OK298497	OK298952	Liu et al. (2022b)
*Trechispora* sp.	DLL2010-077	USA	JQ673209	–	Unpublished
*Trechispora* sp.	DLL2011-186	USA	KJ140681	–	Unpublished
*Trechispora* sp.	F909645	Sweden	JX392817	JX392818	Telleria et al. (2013)
*Trechispora* sp.	He 3431	China	OM523509	OM339316	Liu et al. (2022a)
*Trechispora* sp.	He 3984	China	OM523510	OM339317	Liu et al. (2022a)
*Trechispora* sp.	He 3996	China	OM523511	–	Liu et al. (2022a)
*Trechispora* sp.	He 4503	China	OM523512	OM339318	Liu et al. (2022a)
*Trechispora* sp.	He 5812	Sri Lanka	OM523514	OM339320	Liu et al. (2022a)
*Trechispora* sp.	He 6400	Malaysia	OM523515	OM339321	Liu et al. (2022a)
*Trechispora* sp.	KHL 10715	–	AF347088	AF347088	Larsson et al. (2004)
*Trechispora* sp.	KHL 16968	Brazil	MH290763	–	Chikowski et al. (2020)
*Trechispora* sp.	LWZ 20170805-15	China	OM523517	–	Liu et al. (2022a)
*Trechispora* sp.	LWZ 20170815-20	China	OM523518	OM339322	Liu et al. (2022a)
*Trechispora* sp.	LWZ 20171015-17	Vietnam	OM523519	OM339323	Liu et al. (2022a)
*Trechispora* sp.	LWZ 20180512-12	Australia	OM523520	OM339324	Liu et al. (2022a)
*Trechispora* sp.	LWZ 20180513-8	Australia	OM523521	OM339325	Liu et al. (2022a)
*Trechispora* sp.	LWZ 20180517-43	Australia	OM523522	OM339326	Liu et al. (2022a)
*Trechispora* sp.	LWZ 20180517-44	Australia	OM523523	OM339327	Liu et al. (2022a)
*Trechispora* sp.	LWZ 20180517-45	Australia	OM523524	OM339328	Liu et al. (2022a)
*Trechispora* sp.	LWZ 20191206-27	Malaysia	OM523526	OM339330	Liu et al. (2022a)
*Trechispora* sp.	LWZ 20191208-10	Malaysia	OM523527	–	Liu et al. (2022a)
*Trechispora* sp.	LWZ 20210921-7a	China	OM523530	OM339333	Liu et al. (2022a)
***Trechispora* sp.**	**LWZ 20220829-27a**	**China**	**PV085831**	**PV082941**	**This study**
***Trechispora* sp.**	**LWZ 20220922-4a**	**China**	**PV085833**	**PV082940**	**This study**
***Trechispora* sp.**	**LWZ 20220923-18a**	**China**	**PV085832**	**PV082939**	**This study**
*Trechispora* sp.	NCC16	Brazil	MN701007	MN687968	de Meiras-Ottoni et al. (2021)
*Trechispora* sp.	SP48	Brazil	MN701005	MN687965	de Meiras-Ottoni et al. (2021)
***Trechispora* sp.**	**WEI 16-317**	**Taiwan**	**PV085811**	**PV085821**	**This study**
*Trechispora* sp.	Yuan 6129	China	OM523531	–	Liu et al. (2022a)
*Trechispora* sp.	ZP-3658	China	OM523533	–	Liu et al. (2022a)
*T. stevensonii*	MA-Fungi 70645	–	JX392843	JX392844	Telleria et al. (2013)
*T. subaraneosa*	LWZ 20210918-10a*	China	OM523529	OM339332	Liu et al. (2024)
*T. subconfinis*	LWZ 20230715-12a*	China	PP959670	–	Wang et al. (2024)
*T. subfarinacea*	LWZ 20200921-33a*	China	OM523528	OM339331	Phookamsak et al. (2024)
*T. subfissurata*	He 3907	China	OM523490	OM339298	Liu et al. (2022a)
*T. subhymenocystis*	LWZ 20190818-29b*	China	OM523492	OM339299	Liu et al. (2022a)
*T. subhymenocystis*	LWZ 20190818-32b	China	–	OM339300	Liu et al. (2022a)
*T. subregularis*	VPapp-GF2103	French	OL331097	OL314548	Unpublished
***T. subsinensis***	**GC 2309-119**	**Taiwan**	**PV085815**	**–**	**This study**
*T. subsinensis*	LWZ 20190611-9	China	OM523497	OM339304	Liu et al. (2022a)
***T. subsinensis***	**WEI 19-011**	**Taiwan**	**PV085814**	**–**	**This study**
*T. subsphaerospora*	KHL 8511	Sweden	AF347080	AF347080	Larsson et al. (2004)
*T. taiwanensis*	He 4571*	Taiwan	OM523498	OM339305	Liu et al. (2022a)
*T. termitophila*	AMO396*	Brazil	MN701025	MN687983	de Meiras-Ottoni et al. (2021)
*T. thailandica*	He 4101 *	Thailand	OM523499	OM339307	Liu et al. (2022a)
*T. thelephora*	URM85758	Brazil	–	MH280002	Chikowski et al. (2020)
*T. tongdaoensis*	MHHNU11083*	China	OP959651	OP954662	Deng et al. (2023)
*T. torrendii*	URM85886	Brazil	MK515148	MH280004	Chikowski et al. (2020)
*T. tropica*	LWZ 20170613-14*	China	OM523502	OM339310	Liu et al. (2022a)
*T. tuberculata*	Dai 17433*	Brazil	OM523507	OM339314	Liu et al. (2022a)
*T. wenshanensis*	CLZhao 11649*	China	OQ241389	OQ282716	Luo et al. (2024)
***T. wenshanensis***	**WEI 20-112**	**Taiwan**	**PV085816**	**PV085825**	**This study**
*Tubulicium raphidisporum*	He 3191*	China	OM523534	OM339334	Liu et al. (2022a)

Asterisks (*) indicate holotype sequences. Newly generated sequences are shown in bold

## Results

### Molecular phylogeny

**Fig. 1:**
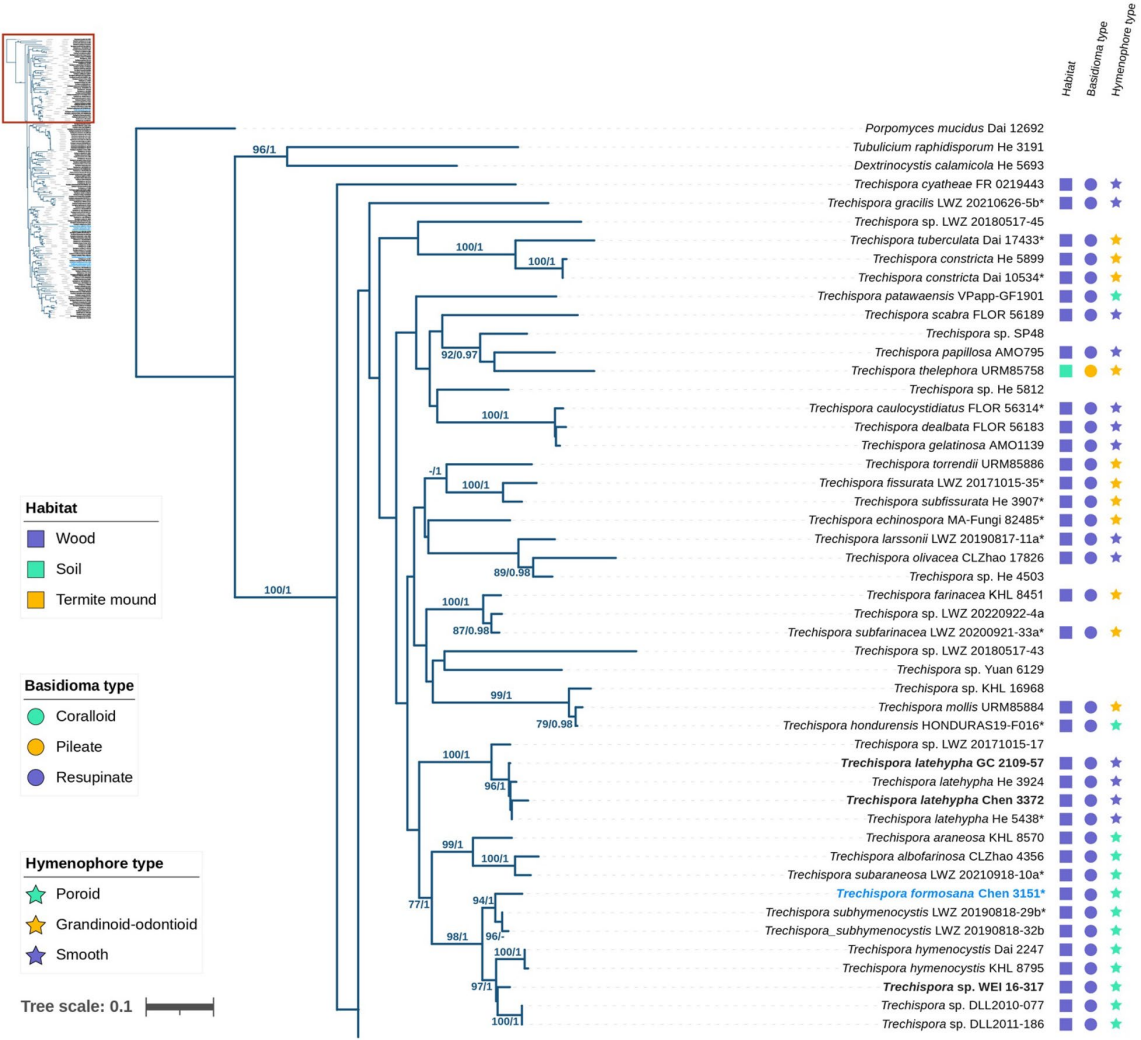

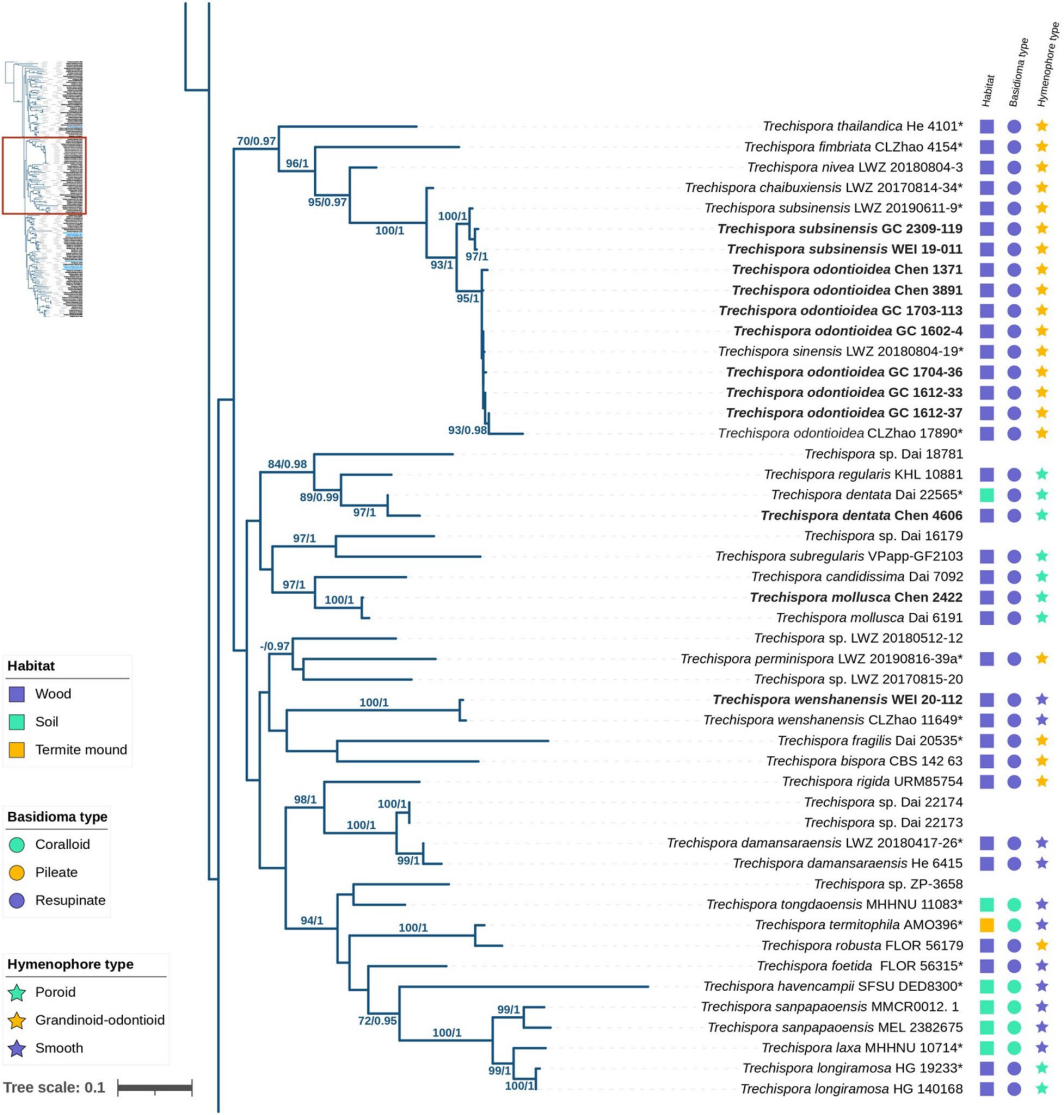

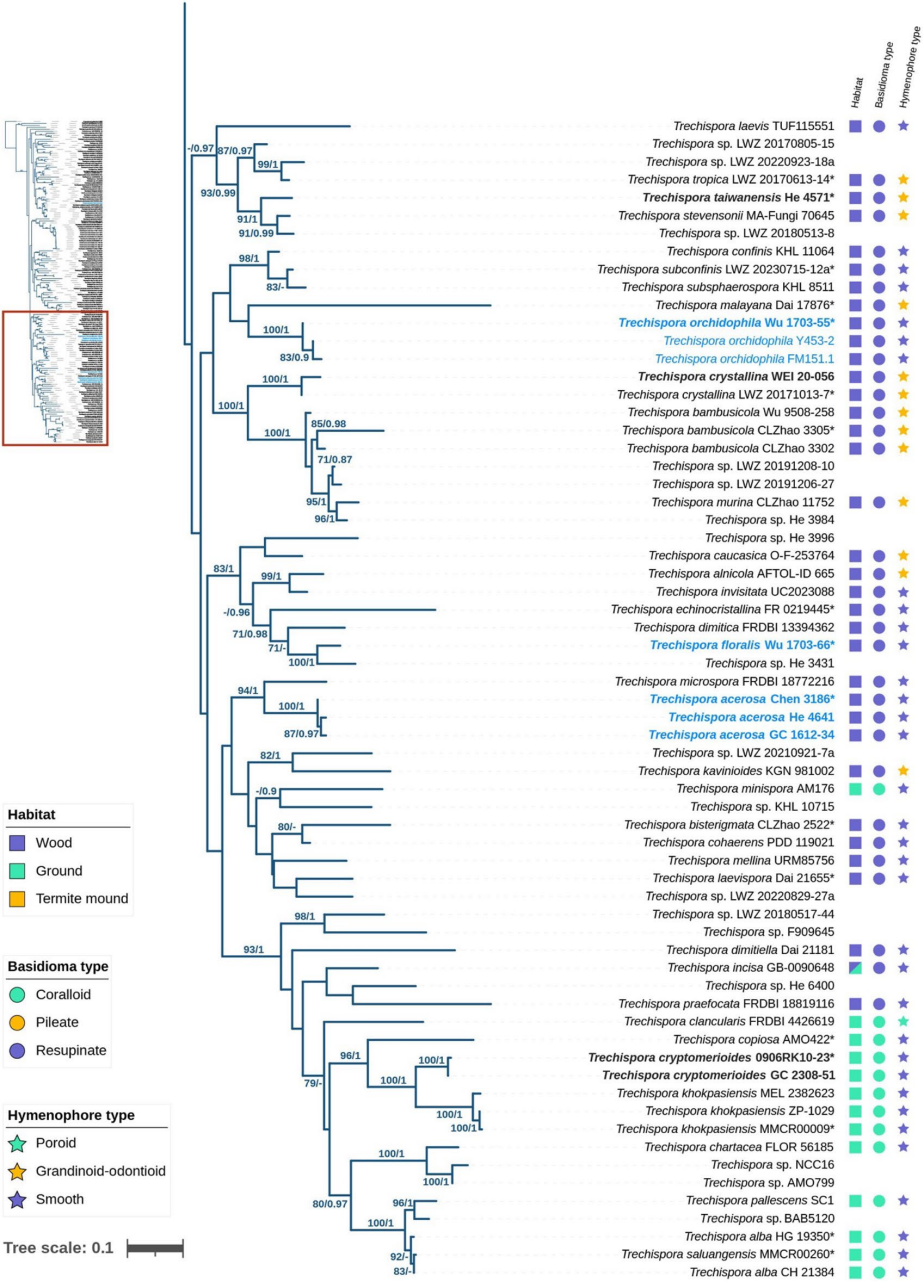
The phylogram of *Trechispora* inferred from ML analyses using ITS + 28S dataset. Branches are labelled with BS ≥ 70% from ML and PP ≥ 0.9 from Bayesian analyses. Blue text indicates new species described in this study, while bold text represents species collected from Taiwan. Asterisks (*) indicate holotype specimens

The ITS + 28S dataset, comprising 162 fungal specimens, was analyzed using Bayesian Inference (BI) and Maximum Likelihood (ML) methods. The alignment contained a total of 2755 sites, including gaps, with 1247 sites for ITS and 1508 for 28S. The General Time Reversible model with gamma rate heterogeneity and invariant sites (GTR + G + I) was identified as the best-fit model for BI analyses based on the Akaike Information Criterion (AIC). The BI and ML analyses yielded similar topologies, supporting the placement of four newly described species (Fig. 1): *Trechispora acerosa*, *T. floralis*, *T. formosana*, and *T. orchidophila* in distinct lineages within the *Trechispora* clade (PP = 1, BS = 100%). *T. acerosa* and *T. orchidophila* formed independent lineages (both PP = 1, BS = 100%) unrelated to other species, while *T. floralis* and *T. formosana* were closely related to *Trechispora* sp. *He 3431* and *T. subhymenocystis*, respectively (PP = 1, BS = 100%; PP = 1, BS = 94%).

### Taxonomy

**Fig. 2:**
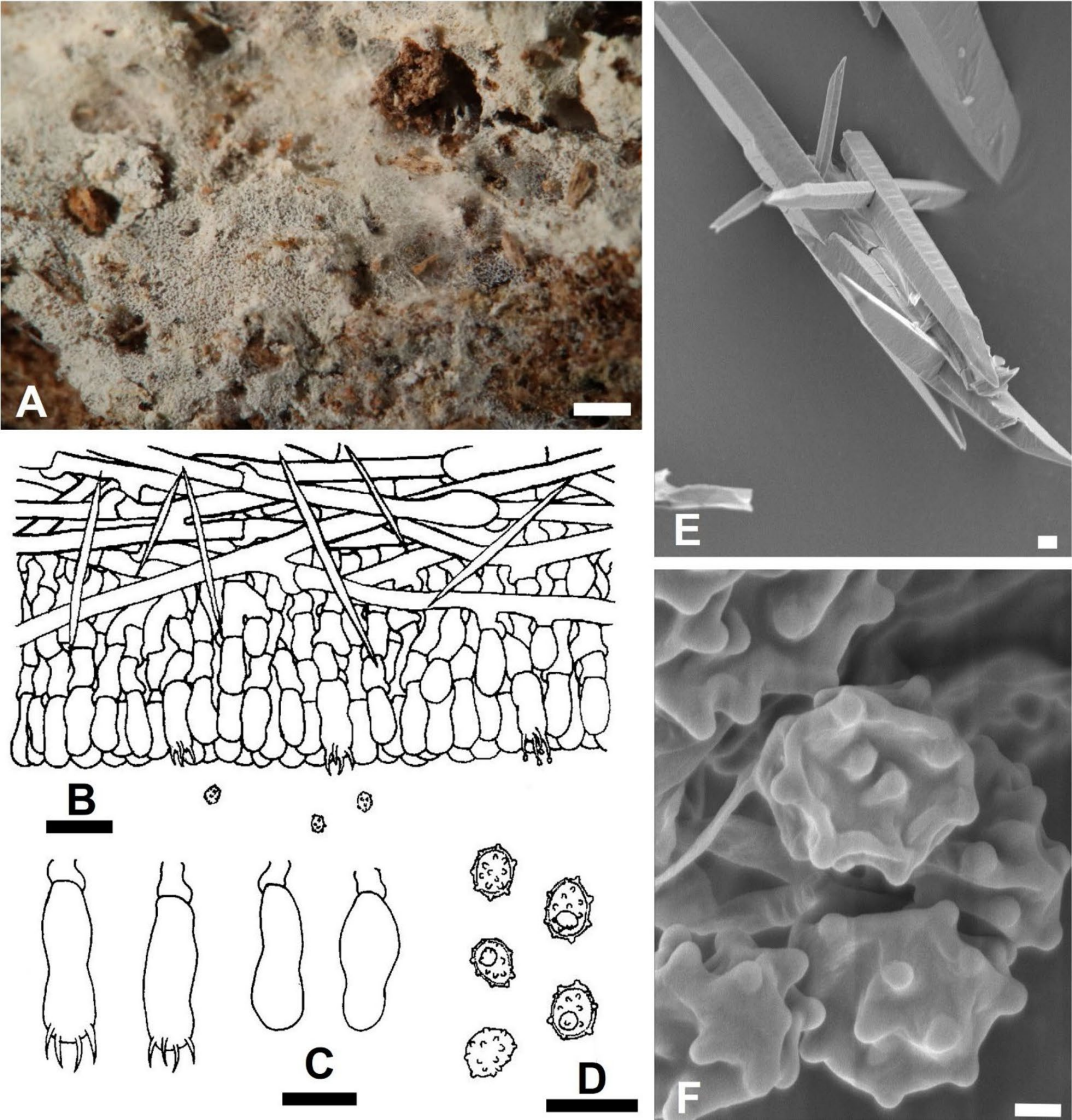
*Trechispora* *acerosa* (from holotype). **A** Basidioma. **B** Part of vertical section through basidioma. **C** Basidia and basidioles. **D** Basidiospores. **E–F** Scanning electron micrographs of needle-like crystals (**E**) and basidiospores (**F**). Scale bars: **A** = 1 mm; **B** = 10 μm; **C–D** = 5 μm; **E** = 1 μm; **F** = 0.5 μm

***Trechispora acerosa*** Yi C. Lin and C. Chih Chen, sp. nov. Figure 2.

MycoBank MB857631.

**Etymology**. *acerosa* (Lat.), referring to the needle-like crystals.

**Diagnosis.** *Trechispora acerosa* is distinguished by its white basidiomata with smooth hymenophore, verrucose, thick-walled basidiospores, and needle-like crystals.

**Typification**. **TAIWAN**. Nantou County, Jenai township, trailhead of Southern Tungyenshan, 24°2'N, 121°6'E, 1550 m, on rotten angiosperm trunk, 28 Mar 2016, leg. S.-Z. Chen, C.-C. Chen & C.-L. Wei, *Chen 3186* (**holotype** TNM F0030009). GenBank: ITS = PV085797; 28S = PV085828.

**Description.** Basidiomata annual, resupinate, thin, soft, fragile, easily separated from substratum, up to 2 cm long, 1 cm wide. Hymenophore smooth, arachnoid to farinose, white to cream. Margin white to cream, fimbriate. Hyphal system monomitic; generative hyphae with clamp connections. Subicular hyphae long-celled, colorless, thin-walled, moderately branched and septate, subparallel to interwoven, 2–5.5 μm in diam, ampullate septa usually present in the hyphae, up to 6 μm wide. Generative hyphae distinct, colorless, thin or slightly thick-walled, short-celled moderately branched, smooth, subparallel, 2.5–4 μm in diam. Needle-like crystals frequently present in subiculum. Cystidia absent. Basidia cylindrical with a slight median constriction, colorless, thin-walled, with four sterigmata and a basal clamp connection, 11–14 × 4–4.6 μm. Basidioles similar in shape to basidia, but smaller. Basidiospores ovoid to ellipsoid, colorless, slightly thick-walled, becoming thick-walled when aged, verrucose, inamyloid, indextrinoid, usually with one oil drop, (2.9–)3.3–3.9(–4.3) × (2.2–)2.4–2.9(–3.2) μm, L = 3.6 μm, W = 2.7 μm, Q = 1.2–1.5 (n = 30).

**Additional specimens examined. TAIWAN**. Nantou County, Luku township, Hsitou, 23°41'N, 120°48'E, 1250 m, on fallen gymnosperm trunk, 11 Dec 2016, leg. S.-Z. Chen & C.-C. Chen, *GC 1612-34* (TNM F0031438); ibid., on rotten *Cunninghamia lanceolata*, 11 Dec 2016, leg. S.-H. He, *He 4641* (BJFC024084). Kaohsiung City, Taoyuan District, Shihshan Forestry Road, 23°04'N, 120°46'E, 1620 m, on rotten angiosperm trunk, 30 Jun 2017, leg. C.-C. Chen, C.-L. Wei, W.-C. Chen and Y.-P. Chen, *WEI 17-439* (TNM F0032508).

**Ecology and distribution.** On rotten angiosperm or gymnosperm (e.g., *Cunninghamia*) trunks in Taiwan. Mar, Jun, Dec.

**Notes.** *Trechispora acerosa* resembles *T. microspora* (P. Karst.) Liberta and *T. praefocata* by having smooth hymenophore, needle-like crystals, and small basidiospores. However, *T. acerosa* differs from *T. microspora* by having thicker and narrower basidiospores (*T. acerosa*: 2.4–2.9 μm; *T. microspora*: 3–3.5 µm) (Bernicchia and Gorjón 2010). A key diagnostic feature of *T. microspora* is the absence of warts near the apiculus of basidiospores (Larsson 1992), whereas in *T. acerosa*, the warts are uniformly distributed (Figs. 2D, F). Compared with *T. praefocata*, *T. acerosa* has narrower basidia (*T. acerosa*: 4–4.6 μm; *T. praefocata*: 5–6 µm) and smaller basidiospores (*T. praefocata*: 5.5–6.5 × 4.5–5 µm) (Liberta 1966).

**Fig. 3:**
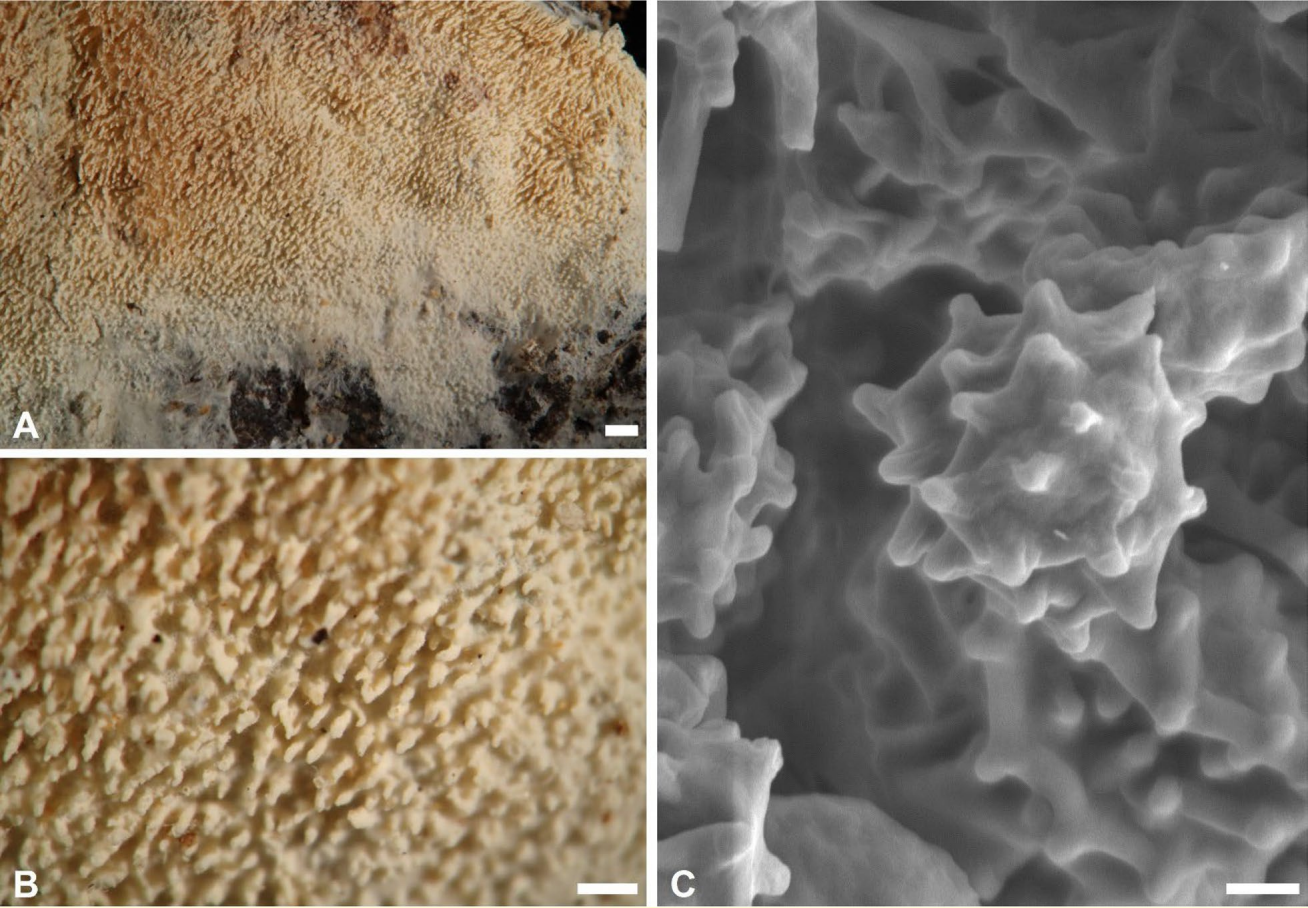
*Trechispora* *crystallina* (from *WEI* *20–056*). **A**–**B** Basidiomata. **C** Scanning electron micrograph of basidiospores. Scale bars: **A** = 1 mm; **B** = 0.5 mm;** C** = 1 μm

***Trechispora crystallina*** S.L. Liu and L.W. Zhou, Mycosphere 13:906. 2022. Figures 3.

**Description and illustration:** See Liu et al. (2022a).

**Specimens examined. CHINA**. Jilin Province, Jilin City, Zuojia Town, 44°4'N, 126°6'E, 270 m, on angiosperm branch, 11 Aug 2016, leg. S.-H. Wu, *Wu 1608-215* (TNM F0030631). **TAIWAN.** Taoyuan City, West Peak of Bajiawanshan, 24°38′08"N, 121°23′25"E, 1690 m, on rotten angiosperm branch, 13 Jul 2020, leg. C.-L. Wei, *WEI 20-056* (TNM F0036784).

**Ecology and distribution.** On living or dead angiosperm wood in China (Inner Mongolia, Jilin), Vietnam, and Taiwan (Liu et al. 2022a; this study). Jul in Taiwan.

**Notes.** *Trechispora crystallina* is characterized by white to cream basidiomata with grandinioid hymenophore, presence of crystals in the subiculum and trama, and verrucose basidiospores (3.5–4.2 × 3–3.6 μm) (Liu et al. 2022a). The examined specimens share these features in most aspects, but *WEI 20-056* has odontioid hymenophore (Figs. 3A–B), and *Wu 1608–215* has basidiospores ranging from verrucose to aculeate. Additional specimens confirm variations in hymenophore configuration (grandinioid to odontioid) and basidiospore ornamentation (verrucose to aculeate) in this species. *Trechispora crystallina* is newly recorded from Taiwan.

**Fig. 4:**
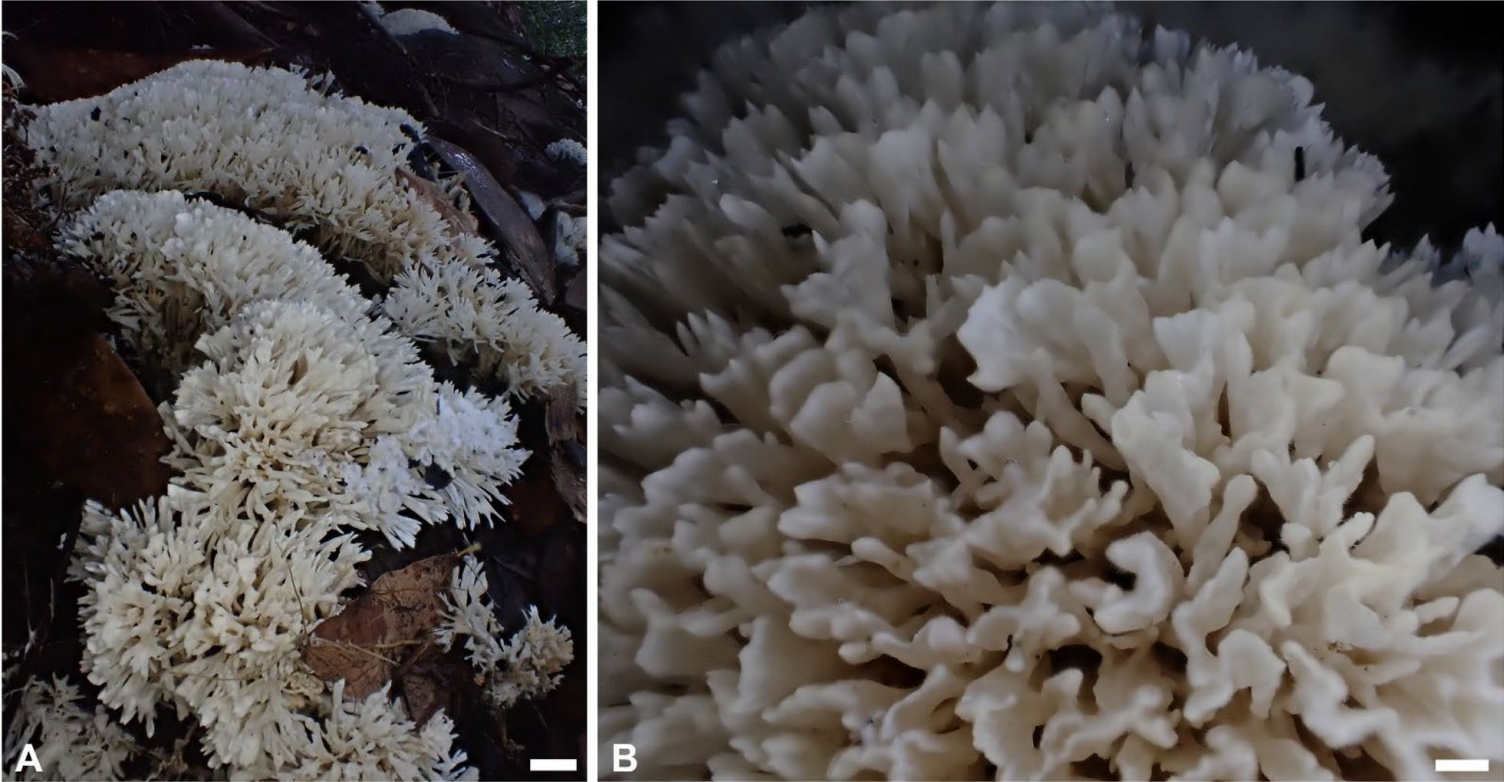
Basidiomata of *Trechispora* *cryptomerioides* (from *GC* *2308–51*) in situ. Scale bars: **A** = 1 cm; **B** = 5 mm. Photographed by Ms. Li-Hung Chen

***Trechispora cryptomerioides*** (W.R. Lin & P.H. Wang) S.L. Liu & L.W. Zhou in Wang et al., Mycology 16:116. 2025. Figure 4.

**Description and illustration.** See Lin et al. (2022, as *Scytinopogon cryptomerioides* W.R. Lin & P.H. Wang).

**Specimens examined. TAIWAN.** Hsinchu County, Jianshi township, Lidong Villa to Mount Lidong Fort, 24°41′29"N, 121°18′23"E, 1500–1900 m, on the ground, 9 Sep 2021, leg. C.-C. Chen, *GC 2109-44* (TNM F0038416); ibid., Mount Lidong Fort, 1900 m, on the ground, 22 Aug 2023, leg. L.-H. Chen, *GC 2308–51* (TNM F0038419); ibid., Syakaro Historic Trail, 24°35′18"N, 121°15′10"E, 1300 m, on the ground, 9 Aug 2023, leg. C.-C. Chen & C.-C. Chang, *GC 2308-12* (TNM F0038418). Nantou County, Hsinyi township, Jenlun Forest Road, on soil, 5 Jul 2009, leg. W.-R. Lin, *0906RK10-23* (TNM F0028829, **holotype**).

**Ecology and distribution.** On the ground in forests dominated by *Cryptomeria japonica* at elevations of 600–2100 m in Taiwan. Jun to Oct. The mycelium is associated with the roots of *C. japonica* (Lin et al. 2022; this study).

**Notes.** *Trechispora cryptomerioides* was originally placed in *Scytinopogon* and was recently transferred to *Trechispora* by Wang et al. (2024) based on morphological and phylogenetic evidence. This species is characterized by white to cream, coralloid basidiomata with smooth hymenophore, and aculeate basidiospores measured as 4–6 × 3–3.5 μm by Lin et al. (2022). Morphology of our specimens agrees with the protologue.

**Fig. 5:**
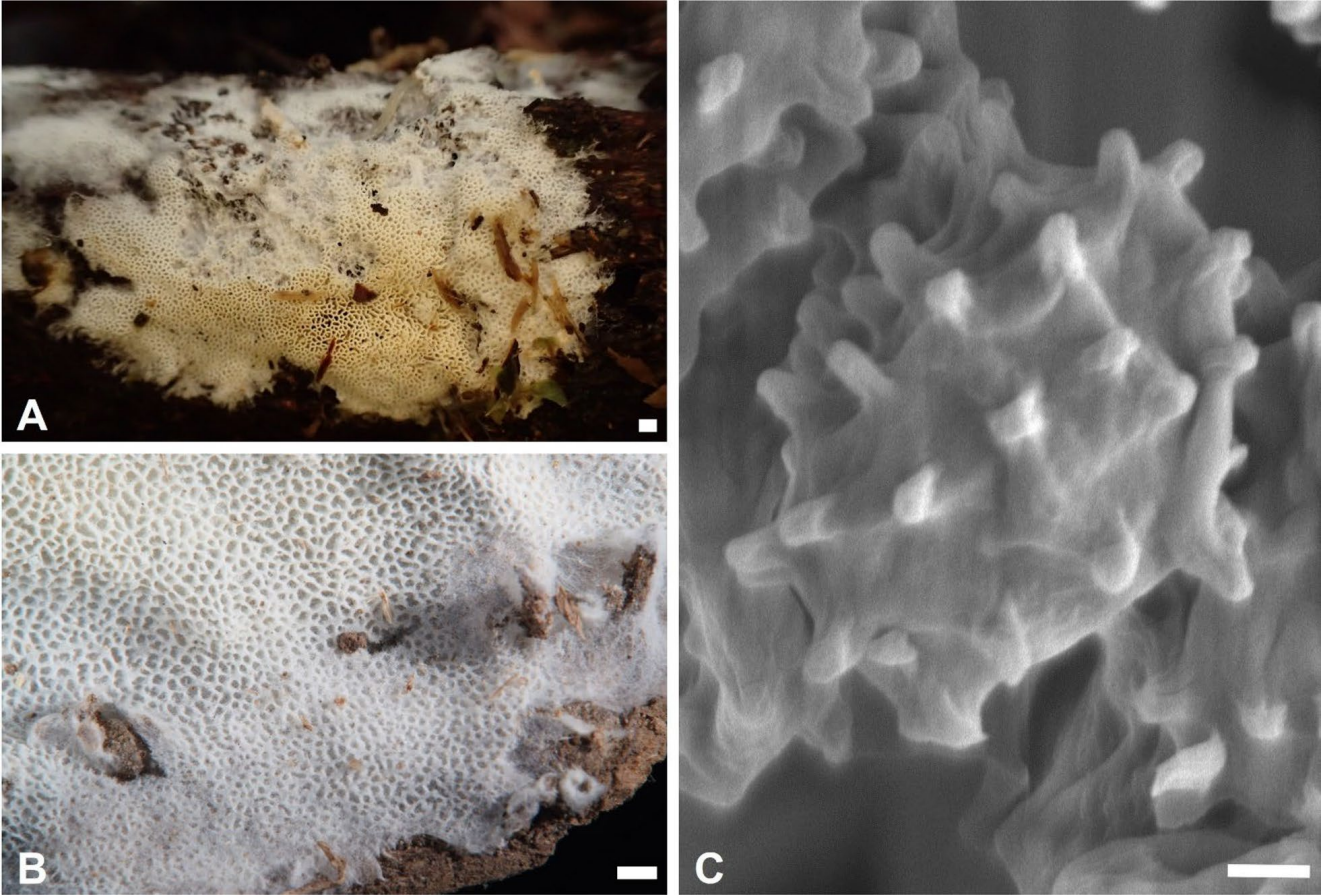
*Trechispora* *dentata* (from *Chen* *4606* except for **A**, which is from *GC* *2405–27*). **A–B** Basidiomata when fresh (**A**) and dry (**B**).** C** Scanning electron micrograph of basidiospores. Scale bars: **A–B** = 1 mm; **C** = 0.5 μm

***Trechispora dentata*** Z.B. Liu & Yuan Yuan, Frontiers in Microbiology 13:9, 2022. Figure 5.

**Description and illustration.** See Liu et al. (2022b).

**Specimens examined. TAIWAN.** Nautou County, Jenai township, Meifeng, 24°05′29''N, 121°10′34''E, 2000–2100 m, on rotten wood, 5 May 2024, leg. C.-C. Chen, *GC 2405-27* (TNM F0038421). Taichung City, Heping District, near Mt. Baimao trailhead, 24°10′10"N, 120°55′10"E, 960 m, on rotten angiosperm wood, 12 May 2021, leg. S.-Z. Chen & C.-L. Wei, *Chen 4606* (TNM F0038415).

**Ecology and distribution.** On soil or rotten angiosperm wood in SW China (Yunnan) and Taiwan (Liu et al. 2022b; this study). May in Taiwan.

**Notes.** *Trechispora dentata* was recently described from Yunnan, China, based on a specimen collected from soil. It is characterized by white to cream basidiomata with distinctly irpicoid to dentate hymenophore (pores or aculei 3–4 per mm), and thick-walled, aculeate basidiospores measured as 4.1–5 × 3.2–4 μm by Liu et al. (2022b). Taiwanese specimens found on rotten wood agree with the protologue, except for having poroid hymenophore with more entire dissepiments (Figs. 5A–B). *T. dentata* is newly recorded from Taiwan.

**Fig. 6:**
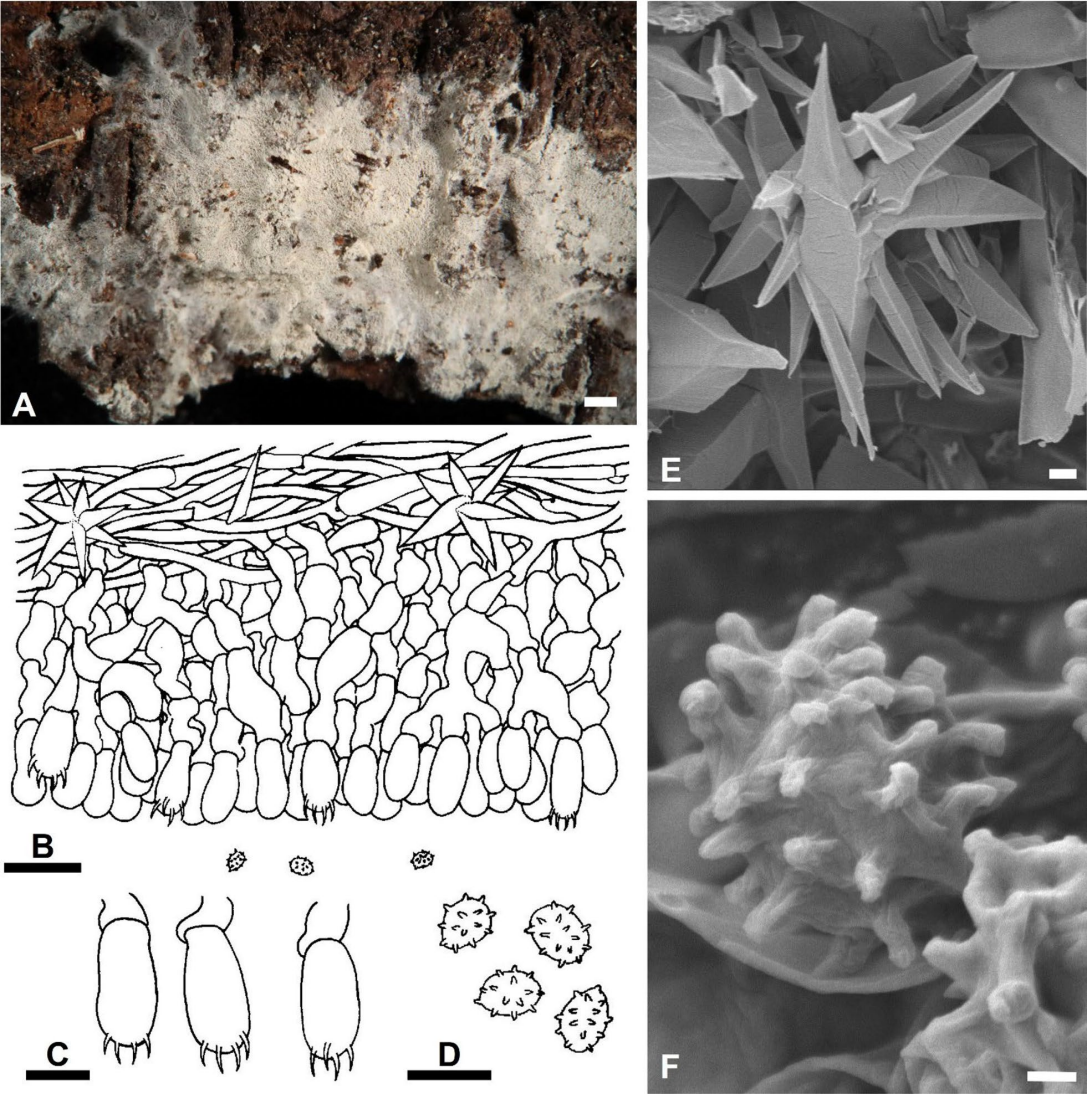
*Trechispora* *floralis* (from holotype). **A** Basidioma. **B** Part of vertical section through basidioma. **C** Basidia. **D** Basidiospores. **E–F** Scanning electron micrographs of flower-like crystals (**E**) and basidiospores (**F**). Scale bars: **A** = 1 mm; **B** = 10 μm; **C–D** = 5 μm; **E** = 1 μm; **F** = 0.5 μm

***Trechispora floralis*** Yi C. Lin & C. Chih Chen, sp. nov. Figure 6.

MycoBank MB857633.

**Etymology.** *floralis* (Lat.), referring to the flower-like crystals.

**Diagnosis.** *Trechispora floralis* is distinguished by its white basidiomata with smooth hymenophore, aculeate basidiospores, and flower-like crystals.

**Typification*.* TAIWAN**. Nantou County, Jenai township, Huisun Forestry Station, 24°5'N, 121°2'E, 750 m, on rotten wood, 24 Mar 2017, leg. S.-H. Wu, *Wu 1703-66* (**holotype** TNM F0031286). GenBank: ITS = PV085813; 28S = PV085822.

**Description.** Basidiomata annual, resupinate, thin, soft, fragile, easily separated from substratum, up to 1.5 cm long, 1 cm wide. Hymenophore smooth to grandinioid, farinaceous, white. Margin white, fimbriate. Hyphal system monomitic; generative hyphae with clamp connections. Subicular hyphae long-celled, colorless, thin-walled, moderately branched and septate, subparallel to interwoven, 1.5–2.5 μm in diam, ampullate septa usually present in the hyphae, up to 4.5 μm wide. Subhymenial hyphae distinct, colorless, tortuous, thin-walled, much branched, smooth, 3.5–7.5 μm in diam. Crystals frequently occurring in subiculum as aggregated, flower-like forms. Cystidia absent. Basidia subclavate to cylindrical, colorless, thin-walled, with four sterigmata and a basal clamp connection, 11–15 × 4.7–5.4 μm. Basidioles similar in shape to basidia, but smaller. Basidiospores ellipsoid, colorless, thin-walled, aculeate, inamyloid, indextrinoid, (3.9–)4.1–4.8(–5.4) × (2.7–)3.1–3.8(–4.2) μm, L = 4.5 μm, W = 3.6 μm, Q = 1.2–1.6 (n = 30).

**Additional specimen examined. TAIWAN**. Nantou County, Jenai township, Aowanda National Forest Recreation Area, 23°57'N, 121°11'E, 1250 m, 28 Aug 2017, on angiosperm branch, leg. C.-L. Wei & Y.-L. Huang, *WEI 17-633* (TNM F0032638).

**Ecology and distribution.** On angiosperm wood in Taiwan. Mar, Aug.

**Notes.** *Trechispora floralis* resembles *T. minima* K.H. Larss. and *T. damansaraensis* S.L. Liu & L.W. Zhou having white basidiomata with smooth hymenophore, and aculeate basidiospore*s.* However, *T. floralis* differs by having flower-like crystals and longer basidiospores than *T. minima* and *T. damansaraensis,* respectively (*T. minima*: 3.5–4 × 3.2–3.7 μm; *T. damansaraensis*: 3–3.8 × 2.3–3 µm) (Larsson 1992; Liu et al. 2022a).

**Fig. 7:**
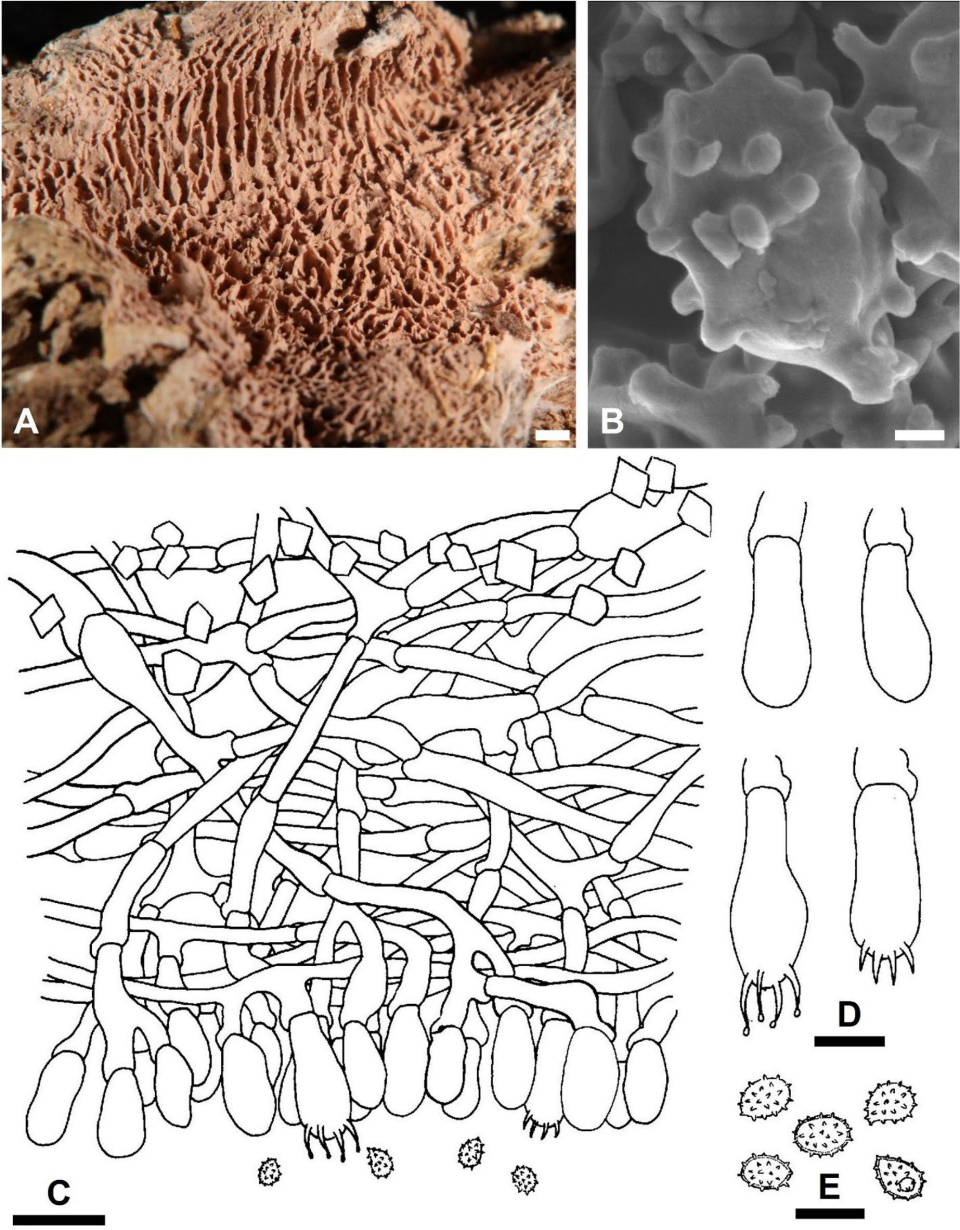
*Trechispora* *formosana* (from holotype except for **A**, which is from *Chen* *3228*). **A** Basidioma. **B** Scanning electron micrograph of basidiospores.** C** Part of vertical section through basidioma. **D** Basidia and basidioles. **E** Basidiospores. Scale bars: **A** = 1 mm; **B** = 0.5 μm; **C** = 10 μm; **D–E** = 5 μm

***Trechispora formosana*** Yi C. Lin & C. Chih Chen, sp. nov. Figure 7.

MycoBank MB857634.

**Etymology.** *formosana* (Lat.), referring to the Formosa, the historical name of Taiwan, the type locality.

**Diagnosis.** *Trechispora formosana* is distinguished by its brick-red basidiomata with poroid hymenophore, and aculeate basidiospores.

**Typification. TAIWAN.** Nantou County, Jenai township, trailhead of Southern Tungyenshan, 24°2'N, 121°6'E, 1550 m, on angiosperm wood, 28 Mar 2016, leg. S.-Z. Chen, C.-C. Chen & C.-L. Wei, *Chen 3151* (**holotype** TNM F0029980). GenBank: ITS = PV085800; 28S = PV085823.

**Description.** Basidiomata annual, resupinate, soft, fragile, easily separated from substrates, up to 3 cm long, 1.5 cm wide. Hymenophore poroid, brick red, with round, angular to irregular pores, 2–3 per mm; dissepiments thin, entire. Subiculum white, soft corky, thin, about 0.1 mm thick. Tubes clay-pink, soft, up to 1 mm long. Margin white, fimbriate, rhizomorphic. Hyphal system monomitic; generative hyphae with clamp connections. Subicular hyphae long-celled, colorless, thin-walled, moderately branched and septate, subparallel to interwoven, 2–5.5 μm in diam, ampullate septa usually present in the hyphae, up to 8.5 μm wide. Generative hyphae distinct, colorless, thin or slightly thick-walled, moderately branched, smooth, more or less parallel, 3–5 μm in diam. Crystals often present as single rhomboidal plates in subiculum. Cystidia absent. Basidia cylindrical with a slight median constriction, colorless, thin-walled, with four sterigmata and a basal clamp connection, 13–22 × 5–6 μm. Basidioles similar in shape to basidia, but smaller. Basidiospores ovoid to ellipsoid, colorless, slightly thick-walled, thick-walled when aged, aculeate, inamyloid, indextrinoid, occasional with one oil drop, (3.8–)4–4.5(–5) × (3.1–)3.2–3.7(–4.4) μm, L = 4.2 μm, W = 3.5 μm, Q = 1.1–1.4 (n = 30).

**Additional specimen examined. TAIWAN.** Nantou County, Jenai township, trailhead of Southern Tungyenshan, 24°2'N, 121°6'E, 1550 m, on rotten wood, 28 Mar 2016, leg. S.-Z. Chen, C.-C. Chen & C.-L. Wei, *Chen 3228* (TNM F0030028).

**Ecology and distribution.** On angiosperm wood in Taiwan. Mar.

**Notes.** *Trechispora formosana* is similar to *T. hondurensis* Schoutteten & Haelew., *T. hymenocystis* (Berk. & Broome) K.H. Larss.*,* and *T. subhymenocystis* S.-L. Liu, H.-S. Yuan & L.-W. Zhou in having poroid hymenophore and aculeate basidiospores. However, *T. hondurensis* differs by having smaller basidiospores (3.7–3.8 × 2.8–2.9 μm) (Haelewaters et al. 2020), while *T. hymenocystis* has sphaerocysts in cords and larger basidiospores (4.5–5.5 × 3.5–4.5 μm) (Larsson 1994). Additionally, *T. subhymenocystis* differs by having white and slightly dentate pore surface, and growing on gymnosperms (Liu et al. 2022a).

**Fig. 8:**
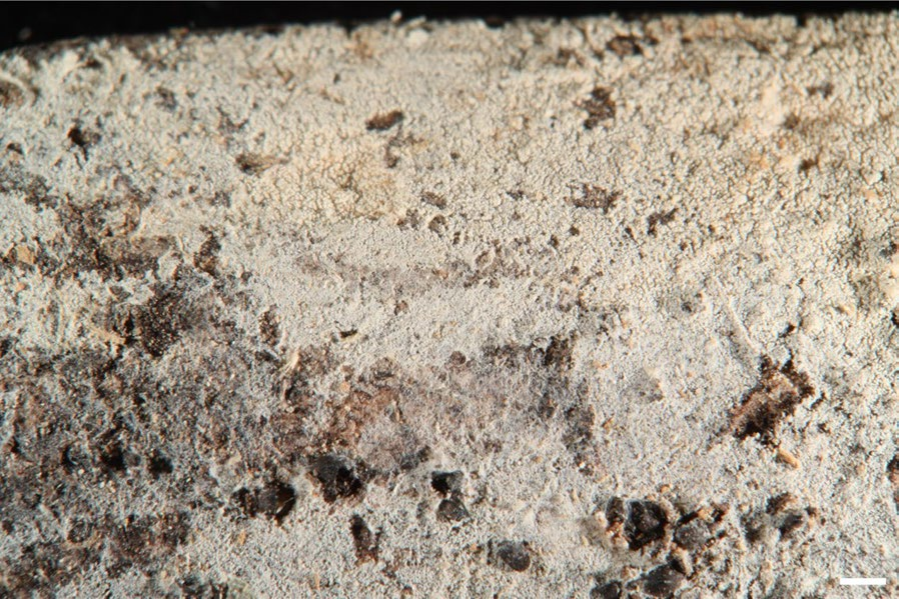
Basidioma of *Trechispora* *latehypha* (from *Chen* *3372*). Scale bar = 1 mm

***Trechispora latehypha*** S.L. Liu, S.H. He & L.W. Zhou, Mycosphere 13:906. 2022. Figure 8.

**Description and illustration.** See Liu et al. (2022a).

**Specimens examined. TAIWAN.** Nautou County, Jenai township, trailhead of Southern Tungyenshan, 24°02'N, 121°06'E, 1568 m, on rotten bamboo culm, 7 Dec 2016, leg. S.-Z. Chen & C.-C. Chen, *Chen 3372* (TNM F0030862). Yilan County, Yuanshan township, Caopi wetland, 24°46′03''N, 121°36′28''E, 800 m, on angiosperm branch, 28 Sep 2021, leg. C.-C. Chen, *GC 2109-57* (TNM F0038417).

**Ecology and distribution.** On angiosperm and gymnosperm wood or bamboo culms in China (Fujian, Guangdong, Hainan) and Taiwan. Jun, Aug, Sep, Dec (Liu et al. 2022a; this study).

**Notes.** *Trechispora latehypha* is characterized by white to cream basidiomata with smooth hymenophore, rows of short-celled, wide hyphae in the subhymenium, thick-walled generative hyphae in the subiculum, and aculeate basidiospores measuring 3–3.5 × 2.4–2.9 μm (Liu et al. 2022a). It resembles *T. wenshanensis* and *T. orchidophila* in having a smooth hymenophore and aculeate basidiospores and may be confused with *T. bambusicola* due to its occurrence on bamboo. However, *T. latehypha* differs from *T. wenshanensis* and *T. orchidophila* by its thick-walled generative hyphae and lack of crystals, while *T. bambusicola* has an odontioid hymenophore. The morphology of our specimens agrees with the protologue. *Trechispora latehypha* is newly recorded from Taiwan.

**Fig. 9:**
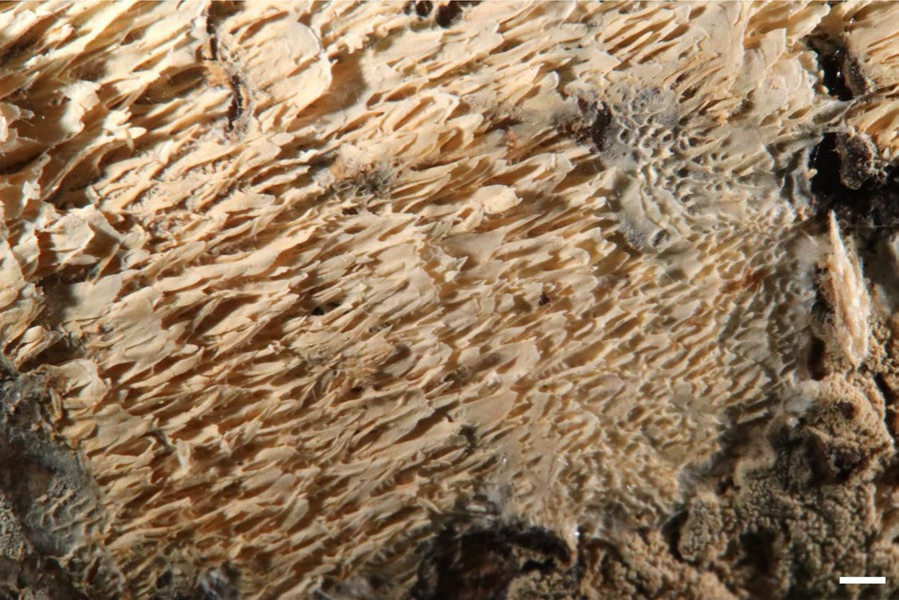
Basidioma of *Trechispora* *mollusca* (from *Chen* *2422*). Scale bar = 1 mm

***Trechispora mollusca*** (Pers.) Liberta, Canadian Journal of Botany 51:1878. 1973. Figure 9.

**Descriptions and illustrations.** See Larsson (1992) and Bernicchia and Gorjón (2010).

**Specimens examined. CHINA.** Jilin Province, Antu County, Changbai Mountain Forest Ecosystem Research Station, 1300 m, 15 Sep 1995, on rotten wood of *Abies*, leg. Y.-C. Dai, *Dai 2154* (TNM F0007320); ibid., 16 Aug 1997, on rotten wood of *Abies*, leg. Y.-C. Dai, *Dai 2565* (TNM F0008167). Liaoning Province, Kuandian County, Baishilazi National Nature Reserve, 1 Sep 2004, on fallen trunk of *Cerasus*, leg. B.-K. Cui, *Cui 1089* (TNM F0017524). **FINLAND.** Kittilän Lappi. Kittila, W of Akäskero, 31 Aug 1999, on fallen trunk of *Betula*, leg. Y.-C. Dai, *Dai 3197* (TNM F0014047). **TAIWAN.** Taichung City, Hoping District, Anma Villa, 24°15'N, 121°0'E, 2150 m, 29 Jul 2014, on rotten angiosperm wood, leg. S.-Z. Chen & W.-C. Chen, *Chen 2422* (TNM F0028196); ibid., Dasyueshan Forestry Road, 1900 m, 19 Aug 2008, on rotten angiosperm branch, leg. S.-H. Wu, S.-Z. Chen & Y.-T. Wang, *Wu 0808-79* (TNM F0022702).

**Ecology and distribution.** On rotten gymnosperm or angiosperm wood in the forest regions of Northern Hemisphere (Larsson 1992; Bernicchia and Gorjón 2010). Jul, Aug in Taiwan.

**Notes.** *Trechispora mollusca* is characterized by white to cream basidiomata with poroid hymenophore (pores 2.5–5 per mm), thick-walled subicular hyphae, and aculeate, subglobose basidiospores measuring 3.5–5 × 2.5–3 μm by Liberta (1973). It resembles *T. dentata* and *T. formosana* in having resupinate basidiomata with poroid hymenophore; however, *T. formosana* has brick-red basidiomata, while *T. dentata* has thin-walled subicular hyphae (Liu et al. 2022b; this study). *Trechispora mollusca* is newly recorded from Taiwan.

**Fig. 10:**
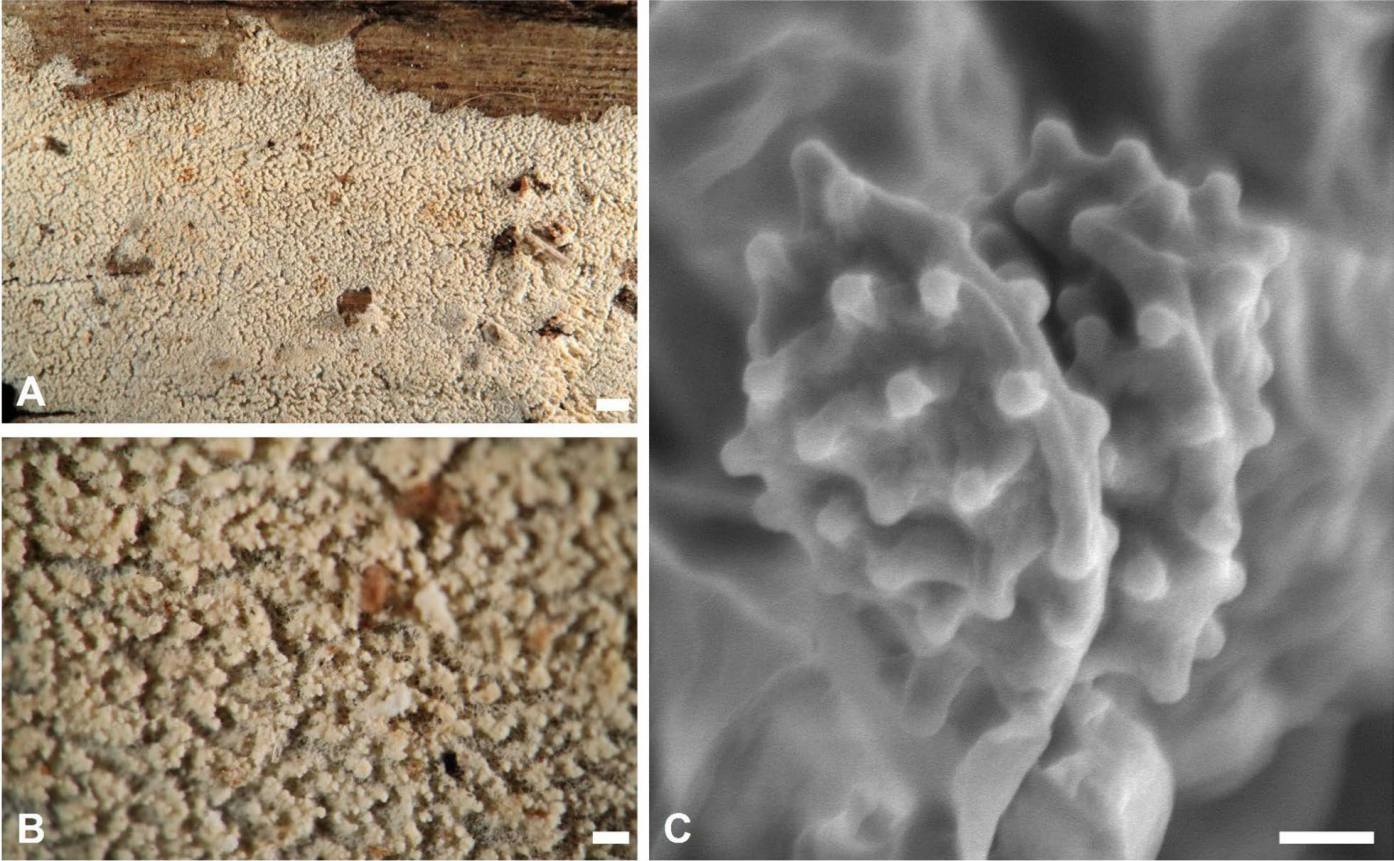
*Trechispora odontioidea* (from *WEI 19–458*). **A**–**B** Basidiomata. **C** Scanning electron micrograph of basidiospores. Scale bars: **A–B** = 1 mm; **C** = 0.5 μm

***Trechispora odontioidea*** K.Y. Luo & C.L. Zhao, Journal of Fungi 8:7. 2022. Figure 10.

 = *Trechispora sinensis* S.L. Liu, L.W. Zhou & S.H. He, Mycosphere 13:924. 2022. (syn. nov.)

**Descriptions and illustrations.** Liu et al. (2022a, as *T. sinensis*) and Luo and Zhao (2022).

**Specimens examined. CHINA.** Jilin Province, Antu County, Erdaobaihe Town, Changbaishan, 42°23'N, 128°6'E, 730 m, on rotten stump, 7 Sep 2011, leg. S.-H. Wu, *Wu 1109-59* (TNM F0025606). Liaoning Province, Dalian City, Jinzhou District, Daheishan, 39°5'N, 121°47'E, 200 m, on rotten angiosperm trunk, 2 Aug 2017, leg. S.-H. Wu, *Wu 1708-78* (TNM F0031640). **TAIWAN.** Kaohsiung City, Maolin District, Tona Nursery, 22°54'N, 120°44'E, 850 m, on angiosperm branch, 31 May 2005, leg. S.-Z. Chen, *Chen 1371* (TNM F0018762). Miaoli County, Taian township, Shei-Pa National Park, East line of Talu Forest Road, 24°30'N, 121°7'E, 2040 m, on rotten trunk, 22 Apr 2017, leg. C.-C. Chen, *GC 1704-36* (TNM F0031482). Nantou County, Hsinyi township, Tungpu, Tsaihung Waterfalls, 23°33'N, 120°56'E, 1400 m, on angiosperm branch, 1 Feb 2016, leg. C.-C. Chen & C.-L. Wei, *GC 1602-4* (TNM F0030309); ibid., Yushan National Park, Japanese Occupation Era Batongguan Historical Trail, 23°33'N, 120°56'E, 1390 m, on fallen angiosperm trunk, 18 Mar 2018, leg. C.-C. Chen, *GC 1803–12* (TNM F0033466); ibid., Jenai township, Aowanda National Forest Recreation Area, 23°57'N, 121°10'E, 1320 m, on angiosperm branch, 13 Sep 2018, leg. C.-L. Wei, *WEI 18–439* (TNM F0034239); ibid., Luku township, Hsitou, 23°41'N, 120°48'E, 1250 m, on angiosperm branch, 11 Dec 2016, leg. S.-Z. Chen & C.-C. Chen, *GC 1612-33* (TNM F0031437); ibid., *GC 1612-37* (TNM F0031441). New Taipei City, Pinglin District, Taweishan Trail, 24°56'N, 121°43'E, 270 m, on angiosperm branch, 23 Mar 2018, leg. S.-Z. Chen, C.-C. Chen & C.-L. Wei, *Chen 3891* (TNM F0033945); ibid., Wanli District, Laoliaohu, 25°10'N, 121°37'E, 470 m, on angiosperm branch, 23 May 2017, leg. Y.-L. Huang, C.-L. Wei & C.-C. Chen, *WEI 17–204* (TNM F0031926); ibid., Wulai District, Neidong National Forest Recreation Area, 24°50'N, 121°32'E, 400 m, on fallen branch, 23 Jun 2011, leg. E.O. Yurchenko, *EYu 110623-28b* (TNM F0024881); ibid., Yangminshan, 25°9'N, 121°33'E, 600 m, on angiosperm branch, 10 Apr 1994, leg. S.H. Wu, *Wu 9404-3* (TNM F0002377). Taichung City, Hoping District, Dasyueshan Forestry Road, 24°15'N, 120°55'E, 1250 m, on bamboo culm, 30 Mar 2017, leg. C.-C. Chen, *GC 1703-113* (TNM F0031212). Taipei City, Peitou District, Tinghushan Hiking Trail, 25°9'N, 121°32'E, 410 m, on angiosperm branch, 2 Dec 2019, leg. C.-L. Wei, *WEI 19-458* (TNM F0036289).

**Ecology and distribution.** On angiosperm branches or trunks, rotten stumps, or bamboo culms in China (Beijing, Chongqing, Fujian, Guangdong, Guangxi, Guizhou, Henan, Hubei, Hunan, Jiangsu, Jiangxi, Jilin, Liaoning, Yunnan) and Taiwan (Liu et al. 2022a; Luo and Zhao 2022; this study). Fruiting year-round and found at elevations ranging from 270 to 2040 m in Taiwan.

**Notes.** *Trechispora sinensis*, recently described from mainland China, is synonymized under *T. odontioidea* based on morphological and phylogenetic evidence (Fig. 1). *T. odontioidea* is characterized by cream to buff basidiomata with odontioid hymenophore, presence of bipyramidal crystals, and thin-walled, verrucose basidiospores measuring 2.8–3.3 × 2.5–2.9 μm (Liu et al. 2022a, b). In Taiwan, *T. odontioidea* is one of the most common *Trechispora* species. It may be confused with *T. bambusicola* C.L. Zhao, which also occurs on bamboo, and *T. subsinensis*. However, *T. bambusicola* differs by having thick-walled basidiospores and lacking crystals (Zhao and Zhao 2021), while *T. subsinensis* differs by having predominantly thick-walled tramal hyphae and aculeate basidiospores (Liu et al. 2022a, b).

**Fig. 11:**
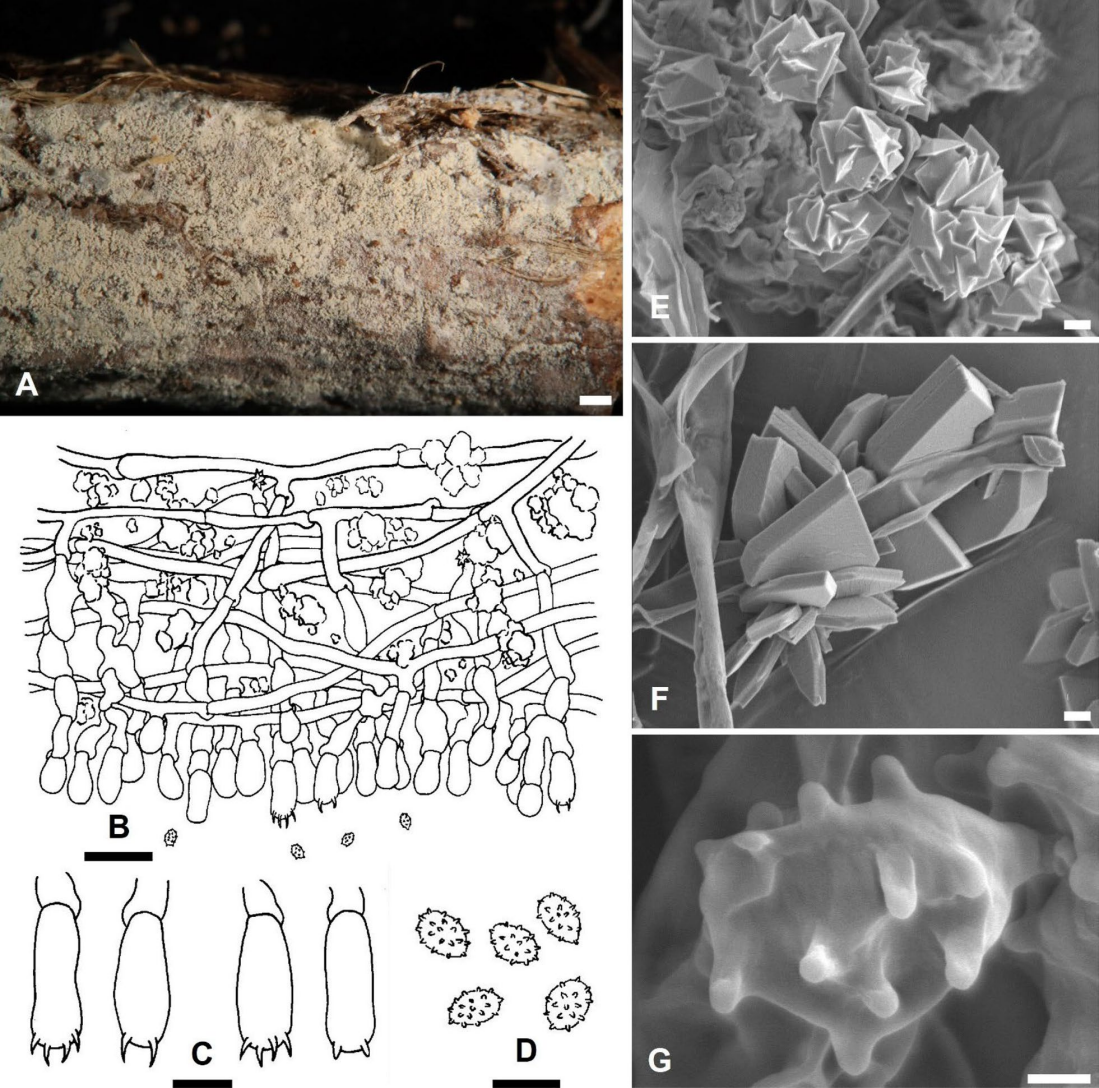
*Trechispora orchidophila* (from holotype). **A** Basidioma. **B** Vertical section through basidioma. **C** Basidia. **D** Basidiospores.** E**–**G** Scanning electron micrographs of crystals in polytetrahedral forms (**E**) and rhomboidal flakes (**F**), as well as basidiospores (**G**). Scale bars: **A** = 1 mm; **B** = 10 μm; **C–D** = 5 μm; **E–F** = 1 μm; **G** = 0.5 μm

***Trechispora orchidophila*** Yi C. Lin & C. Chih Chen, sp. nov. Figure 11.

MycoBank MB857632.

**Etymology.** *orchidophila* (Lat.), referring to its association with orchids.

**Diagnosis.** *Trechispora orchidophila* is characterized by white to cream basidiomata with smooth hymenophore, presence of crystals in subiculum, and thin-walled, aculeate basidiospores.

**Typification. TAIWAN**. Nantou County, Jenai township, Huisun Forestry Station, 24°5'N, 121°2'E, 750 m, on rotten branch, 24 Mar 2017, leg. S.-H. Wu, *Wu 1703–55* (**holotype** TNM F0031278). GenBank: ITS = PV085812; 28S = PV085824.

**Description.** Basidiomata annual, resupinate, thin, soft, fragile, easily separated from substratum, up to 8 cm long, 1 cm wide. Hymenophore smooth, farinaceous, white to cream. Margin white to cream, fimbriate. Hyphal system monomitic; generative hyphae with clamp connections. Subicular hyphae long-celled, colorless, thin-walled, moderately branched and septate, subparallel to interwoven, 2.5–5 μm in diam, ampullate septa usually present in the hyphae, up to 4.5 μm wide. Subhymenium hyphae distinct, colorless, thin-walled, much branched, smooth, 2.5–5 μm in diam. Crystals frequently occurring in subiculum as small, aggregated, polytetrahedral forms or rhomboidal flakes. Cystidia absent. Basidia subclavate to cylindrical, colorless, thin-walled, with two to four sterigmata and a basal clamp connection, 11.4–14.6 × 4.4–4.8 μm. Basidioles similar in shape to basidia, but smaller. Basidiospores ellipsoid, colorless, thin-walled, thick-walled when aged, aculeate, inamyloid, indextrinoid, (3.7–)3.9–4.7(–5.3) × (2.6–)2.9–3.5(–3.8) μm, L = 4.3 μm, W = 3.2 μm, Q = 1.2–1.6 (n = 30).

**Ecology and distribution.** On rotten branch in Taiwan. Mar. Also associated with orchid roots, including Orchidaceae sp. in Réunion (France), and the mycoheterotrophic orchid *Erythrorchis altissima* in Okinawa (Japan) (Martos et al. 2012; Ogura-Tsujita et al. 2018).

**Notes.** The ITS sequences of the holotype *Trechispora orchidophila* (*Wu 1703-55*) has 99% similarity with *FM151.1* (GenBank: JF691276; 573/580) and *Y453-2* (GenBank: LC327027; 574/580), both uncultured environmental samples from orchid roots. *FM151.1* was detected in Orchidaceae sp. in Réunion of France, while *Y453-2* was recovered from *Erythrorchis altissima* in Okinawa of Japan (Martos et al. 2012; Ogura-Tsujita et al. 2018). Phylogenetically, *T orchidophila* is closely related to *T. malayana* S.L. Liu, S.H. He & L.W. Zhou (Fig. 1). However, *T. malayana* has odontioid hymenophore and aculeate basidiospores.

Morphologically, *Trechispora orchidophila* resembles *T. larssonii* S.L. Liu, L.W. Zhou & S.H. He and *T. latehypha* S.L. Liu, S.H. He & L.W. Zhou in having white to cream basidiomata with smooth to grandinioid hymenophore, presence of rhomboidal crystals, and ellipsoid basidiospores. However, *T. orchidophila* differs by having longer basidiospores (*T. larssonii*: 2.8–3.3 μm*; T. cyatheae*: 3–3.5 μm) (Liu et al. 2022a; Ordynets et al. 2015).

**Fig. 12:**
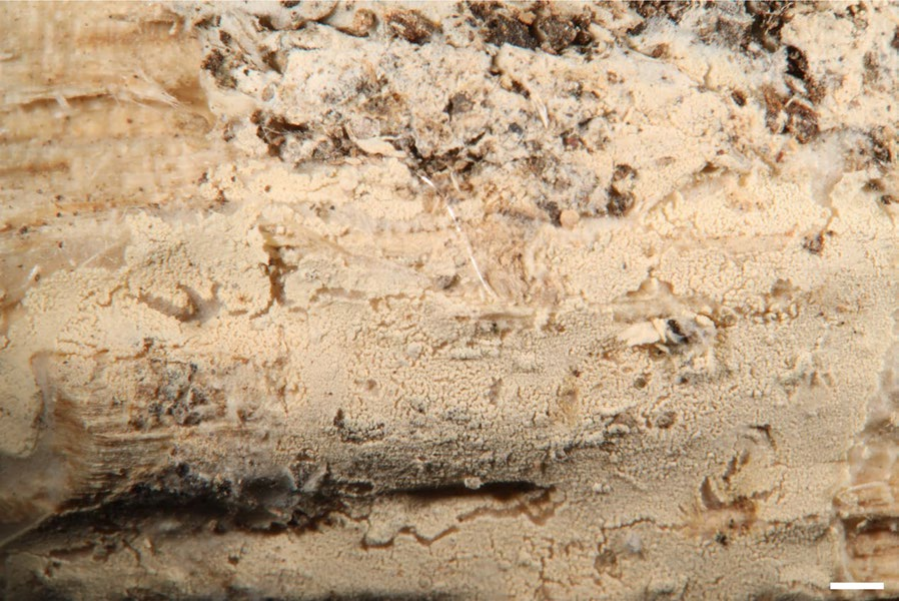
Basidioma of *Trechispora rigida* (from *Wu 0008–5*). Scale bar = 1 mm

***Trechispora rigida*** (Berk.) K.H. Larss., Nordic J. Bot. 16:92. 1996. Figure 12.

**Description and illustration.** Larsson (1996).

**Specimens examined. CHINA**. Liaoning Province, Fushun City, Qingyuan Manchu Autonomous County, Qingyuan ecological experiment station, 41°51'N, 124°56'E, 590 m, on angiosperm branch, 30 Jul 2017, S.-H. Wu, *Wu 1707-127* (TNM F0031564). Yunnan Province, Hsishuangpanna, Green Stone Forest Park, 21°55'N, 101°17'E, 650 m, on branch of angiosperm, 18 Aug 1997, S.-H. Wu & S.-Z. Chen, *Wu 9708–313* (TNM F0007941); ibid., Chuxiong Yi Autonomous Prefecture, Nanhua County, Tujie Town, 24°49'N, 100°46'E, 1915 m, on gymnosperm branch, 15 Aug 2017, C.-C. Chen, *GC 1708–314* (TNM F0032823). **TAIWAN**. Nantou County, Huisun Forestry Station, 700 m, on angiosperm branch, 4 Jun 1999, S.-H. Wu, *Wu 9901–20* (TNM F0010251); ibid., Tungpu village, 23°33'N, 120°56'E, on angiosperm trunk, 20 Sep 1995, 1600 m, S.-H. Wu & S.-Z. Chen, *Wu 9509–26* (TNM F0004256); ibid., Shalihsienhsi Forest Road, 23°32'N, 120°55'E, 1350 m, on angiosperm branch, 24 Nov 1993, leg. S.-H. Wu & S.-Z. Chen, *Wu 9311–78* (TNM F0001504). Pingtung County, Manchou township, Chufengshan, 22°2'N, 120°51'E, 90 m, on angiosperm branch, 29 Nov 2018, C.-C. Chen & C.-L. Wei, *WEI 18–606* (TNM F0034700); ibid., Wanliteshan, 22°4′33"N, 120°50′12"E, 230 m, on angiosperm branch, 29 Nov 2018, C.-C. Chen & C.-L. Wei, *WEI 18–679* (TNM F0034741). Taitung County, Orchid Island, Chungai Bridge, on rotten angiosperm branch, 50 m, 30 Apr 1997, S.-H. Wu & J.Y. Tseng, *Wu 9704-128* (TNM F0008677); ibid., Taimali, 850 m, on angiosperm branch, S.-H. Wu, *Wu 890519-27* (TNM F0014986). Taipei City, Yangminshan National Park, Luchiaokenghsi Ecological Reserve, 25°11'N, 121°34'E, 350 m, on rotten angiosperm branch, 21 Oct 2001, S.-H. Wu et al., *Wu 0008-5* (TNM F0012765).

**Ecology and distribution.** On angiosperm and gymnosperm wood in Argentina, Brazil, China (Liaoning, Yunnan), and Taiwan (Larsson 1996; Hjortstam and Ryvarden 2007; Silveira 2016; this study). Jun, Apr, Sep to Nov in Taiwan.

**Notes.** *Trechispora rigida* is characterized by white to cream basidiomata with grandinioid hymenophore and aculeate, concave basidiospores, originally measured as 4.5–5.5 × 4 μm by Larsson (1996). The examined specimens from mainland China and Taiwan share these morphological features, though their basidiospores were slightly smaller (4–4.5 × 3–3.5 μm). This species was first reported from Taiwan by Wu (2003).

**Fig. 13:**
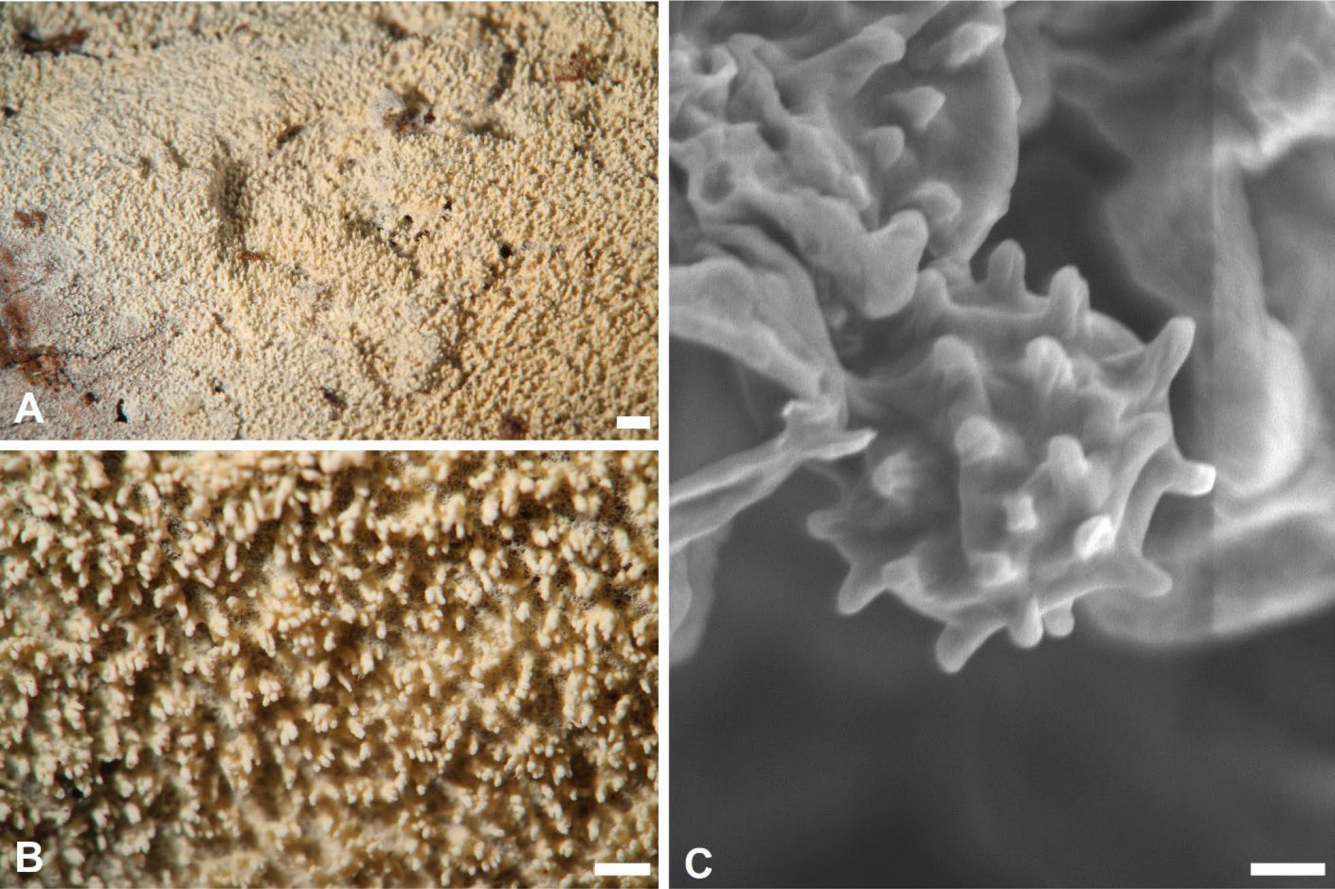
*Trechispora subsinensis* (from *GC 2309–119*). **A**–**B** Basidiomata. **C** Scanning electron micrograph of basidiospores. Scale bars: **A–B** = 1 mm; **C** = 0.5 μm

***Trechispora subsinensis*** S.L. Liu, S.H. He & L.W. Zhou, Mycosphere 13:931. 2022. Figure 13.

**Description and illustration.** See Liu et al. (2022a).

**Specimens examined. CHINA.** Guangxi Province, Dayaoshan Nature Reserve, Hekou Protection Station, 24°8'N, 110°5'E, 620 m, on angiosperm trunk, 9 Sep 2018, leg. S.-H. Wu, *Wu 1809-21* (TNM F0032915). **TAIWAN.** Kaohsiung City, Maolin District, Shan-Ping Forest Ecological Garden, 22°58'N, 120°41'E, 730 m, 29 Jun 2017, on rotten wood, leg. C.-C. Chen, C.-L. Wei, W.-C. Chen & Y.-P. Chen, *WEI 17-359* (TNM F0032470); ibid., on angiosperm branch, leg. C.-C. Chen, C.-L. Wei, W.-C. Chen, & Y.-P. Chen, *WEI 17-401* (TNM F0032489). New Taipei City, Pinghsi District, Lingjiaoliao Mountain Trail, 25°2'N, 121°45'E, 210 m, on stem of *Arenga engleri*, 23 Aug 2018, leg. S.-Z. Chen, C.-C. Chen, & C.-L. Wei, *Chen 3821* (TNM F0033911); ibid., Wulai Distric, Kalamoji Trail, 24°47'N, 121°30'E, 380 m, on angiosperm branch, 23 Jan 2019, leg. C.-C. Chen & C.-L. Wei, *WEI 19-011* (TNM F0034764). Pingtung county, Manchou township, Chufengshan, 22°4'N, 120°51'E, 120 m, on rotten angiosperm trunk, 25 Apr, 2007, leg. S.-H. Wu, S.-Z. Chen & D.-M. Wang, *Wu 0704–14* (TNM F0020980); ibid., Nanjenshan, 22°5'N, 120°52'E, 320 m, on angiosperm branch, 28 Nov 2018, leg. C.-C. Chen & C.-L. Wei, *WEI 18-531* (TNM F0034466). Taichung City, Hoping District, Wuwoweishan, 24°14'N, 120°58'E, 1960 m, on angiosperm branch, 26 Oct 2017, leg. C.-L. Wei & Y.-L. Huang, *WEI 17-717* (TNM F0032718). Taitung county, Orchid Island, 22°00'N, 121°34'E, 87 m, on angiosperm branch, leg. C.-C. Chen, S.-Z. Chen & S.-W. Chou, 22 Sep 2023, *GC 2309-119* (TNM F0038420).

**Ecology and distribution.** On angiosperm (e.g., *Arenga*) branches or trunks in China (Guangdong, Guangxi), Thailand (Chiang Mai), and Taiwan (Liu et al. 2022a; this study). Occurs at elevations from 87 to 1960 m, recorded in Jan, Apr, Jun to Nov in Taiwan.

**Notes.** *Trechispora subsinensis* is newly recorded from Taiwan and is characterized by white to cream basidiomata with an odontioid hymenophore, thick-walled tramal hyphae, and aculeate basidiospores measuring 3–4 × 3–3.5 μm. The basidiospores of the examined specimens in this study are more globose and larger than those of the holotype (2.7–3.5 × 2.3–2.8 μm) (Liu et al. 2022a). Morphologically, *T. subsinensis* resembles *T. crystallina* and *T. odontioidea* due to its grandinioid to odontioid hymenophore and similar basidiospore size. However, *T. crystallina* differs by its thin-walled tramal hyphae and verrucose basidiospores, while *T. odontioidea* is distinguished by its verrucose basidiospores (Liu et al. 2022a).

**Fig. 14:**
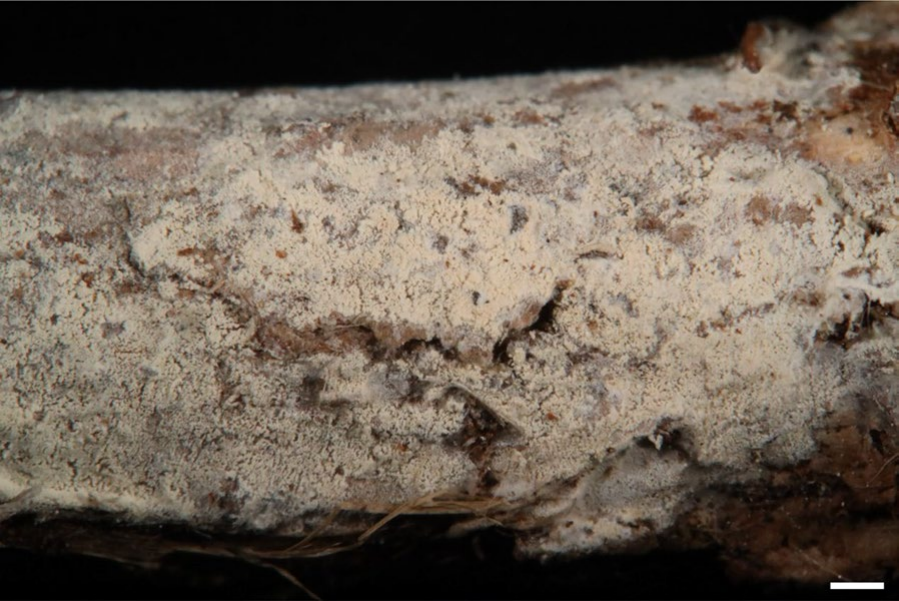
Basidioma of *Trechispora taiwanensis* (from *GC 1703–113*). Scale bar = 1 mm

***Trechispora taiwanensis*** S.L. Liu, S.H. He & L.W. Zhou, Mycosphere 13:934. 2022. Figure 14.

**Description and illustration.** See Liu et al. (2022a).

**Specimens examined. TAIWAN**. Nantou County, Lianhuachi, on dead bamboo, 6 Dec 2016, leg. S.H. He, *He 4571* (BJFC 024012, **holotype**). Taichung City, Hoping District, Dasyueshan Forestry Road. 24°15'N, 120°55'E, 1250 m, on bamboo culm, 30 Mar 2017, leg. C.-C. Chen, *GC 1703-113* (TNM F0031212).

**Ecology and distribution.** On bamboo culm in Taiwan. Mar, Dec (Liu et al. 2022a; this study).

**Notes.** *Trechispora taiwanensis* is characterized by white to cream basidiomata with smooth to grandinioid hymenophore, ellipsoid, aculeate basidiospores measuring 3–4 × 2–2.8 μm (Liu et al. 2022a). It may be confused with *T. latehypha* and *T. odontioidea*, which also occur on bamboo. However, *T. latehypha* differs by its smooth hymenophore and thick-walled generative hyphae, while *T. odontioidea* has an odontioid hymenophore and verrucose basidiospores (Liu et al. 2022a).

**Fig. 15:**
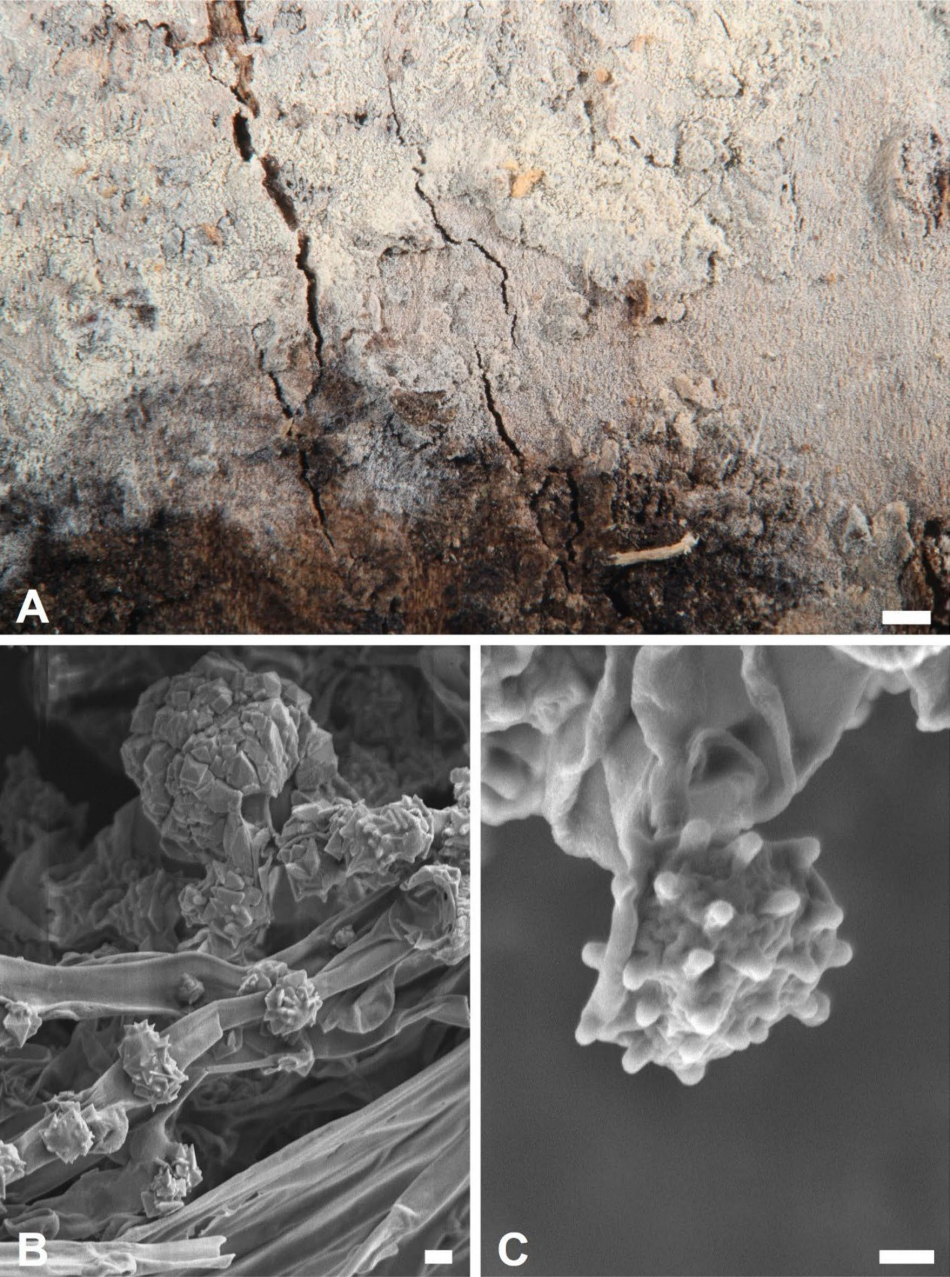
*Trechispora wenshanensis* (from *WEI 20–112*). **A** Basidioma. **B**–**C** Scanning electron micrographs of irregular crystals (**A**) and basidiospores (**B**). Scale bars: **A** = 1 mm; **B** = 1 μm **C** = 0.5 μm

***Trechispora wenshanensis*** K.Y. Luo & C.L. Zhao, MycoKeys 105:167. 2024. Figure 15.

**Description and illustration.** See Luo et al. (2024).

**Specimen examined. TAIWAN.** Taoyuan City, Fuxing District, on the way to Sileng Hot Spring, 24°38'N, 121°26'E, 1140 m, on angiosperm trunk, 12 Jul 2020, leg. C.-L. Wei, *WEI 20-112* (TNM F0038414).

**Ecology and distribution.** On angiosperm branches and trunks in SW China (Yunnan) and Taiwan. Jul in Taiwan.

**Notes.** *Trechispora wenshanensis* is characterized by cream to buff basidiomata with smooth hymenophore, narrow generative hyphae (1–2 μm in diam), and aculeate basidiospores measuring 2.5–3.7 × 2–3 μm (Luo et al. 2024). The Taiwanese specimen has longer basidiospores (3.6–4.2 μm) and small, aggregated, irregular crystals in subhymenium (Fig. 15B). *Trechispora wenshanensis* is newly recorded from Taiwan.

### Key to known species of *Trechispora* from Taiwan

1. Basidiomata clavarioid, on ground…*Trechispora cryptomerioides *
  Basidiomata resupinate, on wood…2
2. Hymenophore poroid…3
  Hymenophore non-poroid…5
3. Hymenial surface white ivory pale yellow, subicular hyphae slightly thick-walled, up to 3 μm wide…4
  Hymenial surface brick-red, subicular hyphae thin-walled, up to 6 μm wide…*T. formosana*
4. Basidiospores subglobose, < 4 μm long…*T. mollusca *
  Basidiospores ellipsoid, > 4 μm long…*T. dentata*
5. Hymenophore smooth…6
  Hymenophore non-smooth…12
6. Hyphal system dimitic…*T. dimitica *
  Hyphal system monomitic…7
7. Crystals in subiculum acicular…8
  Crystals in subiculum differently shaped…9
8. Basidiospore including spines > 5 μm long, > 4 μm wide…*T. praefocata *
  Basidiospore < 4 μm long, < 3 μm wide…*T acerosa*
9. Crystals petaliform, basidiospores 4.1–4.8 × 3.1–3.8 μm…*T. floralis *
  Crystals in shape of rhomboidal flakes or absent…10
10. Subhymenial hyphae thick-walled…*T. latehypha *
  Subhymenial hyphae thin-walled…11
11. Subhymenial hyphae < 2 μm wide…*T. wenshanensis *
  Subhymenial hyphae > 2 μm wide…*T. orchidophila*
12. Hymenophore colliculose or grandinioid; tramal hyphae thin-walled or slightly thick-walled…13
  Hymenophore odontoid to hydnoid; tramal hyphae distinctly thick-walled…16
13. On bamboo…*T. taiwanensis *
  On other substrata…14
14. Basidiospores ventrally concave…*T. rigida. *
  Basidiospores ventrally straight or convex…15
15. Basidiospores verrucose…*T. crystallina *
  Basidiospores aculeate…*T. farinacea*
16. Basidiospores verrucose…*T. odontioidea*
  Basidiospores aculeate…*T. subsinensis*

## Discussion

### Taxonomy and diversity of *Trechispora* in Taiwan

Taxonomic studies on the genus *Trechispora* have traditionally focused on Europe and the Americas (Karsten 1890; Larsson 1992, 1996; Chikowski et al. 2020), with more recent research expanding into the Indo-Pacific region (Ordynets et al. 2015; Liu et al. 2022a; Sommai et al. 2023). This study provides the first comprehensive revision of *Trechispora* in Taiwan, integrating morphological and molecular data.

Prior to this study, only seven species had been recorded in Taiwan (Lin et al. 2022; Liu et al. 2022a; Maekawa 1992; Wu 2003), but molecular data were unavailable for some of them, leaving their phylogenetic placements uncertain. We confirmed the taxonomic status of *T. cryptomerioides*, *T. rigida*, and *T. taiwanensis* based on morphological re-examinations, while *T. lunata* was previously transferred to *Sidera* as *S. lunata* (Romell ex Bourdot & Galzin) K.H. Larss. (Miettinen and Larsson 2011). However, Taiwanese *T. rigida* remains phylogenetically unresolved due to the lack of molecular data. *T. dimitica*, *T. farinacea*, and *T. praefocata*, originally reported from Orchid Island, were not recollected in this study, and their current distribution requires further investigation.

This study expands the known diversity of *Trechispora* in Taiwan, increasing the number of recognized species to 17. Four new species (*T. acerosa*, *T. floralis*, *T. formosana*, and *T. orchidophila*) are described, and seven species are newly recorded for Taiwan (*T. crystallina*, *T. dentata*, *T. latehypha*, *T. mollusca*, *T. odontioidea*, *T. subsinensis*, and *T. wenshanensis*). These findings highlight Taiwan as a previously underexplored region for *Trechispora* and suggest that additional undiscovered species may be present.

### Ecological significance of *Trechispora* in Taiwan

Our findings indicate that *Trechispora* species in Taiwan inhabit a broad range of substrates, including dead or living angiosperm and gymnosperm wood, as well as soil. Among them, *T. odontioidea* and *T. subsinensis* are the most frequently encountered species, occurring year-round on angiosperm wood and bamboo culms from lowland areas to around 2000 m. The ecological role of *T. dentata*, originally described from soil in Yunnan (Liu et al. 2022b) but found in Taiwan on highly decomposed wood, remains unclear. A similar pattern is observed in *T. incisa* (Larsson 1992), suggesting that these species may not function as true wood-decay fungi but rather as soil- and wood-inhabiting fungi with alternative ecological strategies.

A unique case is *T. cryptomerioides*, the only known coralloid *Trechispora* species in Taiwan. Unlike other species that primarily inhabit wood, *T. cryptomerioides* is found on the ground in *Cryptomeria japonica*-dominated forests at 1300–1950 m. DNA of *T. cryptomerioides* has been detected in *C. japonica* root systems (Lin et al., unpublished data), and its fruiting is influenced by stand thinning (Lin et al. 2015). These findings suggest a close association between *T. cryptomerioides* and *C. japonica*, raising the possibility of a mycorrhizal relationship, though further studies are required to confirm this interaction (Lin et al. 2022).

Another noteworthy species is *T. orchidophila*, which was collected from rotten wood but is phylogenetically linked to environmental samples from orchid roots in Réunion (France) and Okinawa (Japan) (Martos et al. 2012; Ogura-Tsujita et al. 2018). This suggests that *T. orchidophila* may function as a saprotroph or root endophyte, potentially forming symbiotic associations with orchids, although additional studies are needed to clarify its ecological role.

Although *Trechispora* species are known to associate with termites, no termite-associated species have been recorded in Taiwan. In other regions, *T. polygonospora* Ryvarden and *T. termitophila* Meiras-Ottoni & Gibertoni have been found in termite mounds (de Meiras-Ottoni et al. 2021; Larsson 1992), and certain species produce termite egg-mimicking structures (termite balls) within termite nests (Matsuura and Yashiro 2010). Future surveys of termite nests and decomposing wood in Taiwan’s tropical and subtropical forests may reveal new termite-associated *Trechispora* species.

## Data Availability

Specimens have been deposited at the fungaria of BJFC and TNM. Newly described species has been registered at MycoBank. DNA sequences have been deposited at GenBank. The files of sequence alignments have been deposited in the Figshare depository (http://dx.doi.org/10.6084/m9.figshare.28407632).
